# Development and Validation of a Multimodal–Multitask Deep Learning Approach for Estimating Late Distant Recurrence Risk in HR-Positive Early Breast Cancer

**DOI:** 10.1158/2767-9764.CRC-26-0362

**Published:** 2026-07-31

**Authors:** Eleftherios P. Mamounas, Ming Chen, Joseph A. Sparano, Md Ashequr Rahman, Yating Cheng, Victoria Wang, Robert J. Gray, Priya Rastogi, Eghbal Amidi, Charles E. Geyer, Tommy Boucher, Tanner J. Freeman, Mohammadreza Ramzanpour, Mukund Varma, Hassan Ghani, Caleb Cheng, Casey Bales, Jennifer R. Ribeiro, Hanna Bandos, Nicolas Stransky, Mark R. Miglarese, Matthew Oberley, David Spetzler, Milan Radovich, George W. Sledge, Norman Wolmark

**Affiliations:** 1 NSABP Foundation, NRG Oncology, Pittsburgh, Pennsylvania.; 2 AdventHealth Cancer Institute, Orlando, Florida.; 3 Caris Life Sciences, Phoenix, Arizona.; 4 Icahn School of Medicine at Mount Sinai, Tisch Cancer Institute, New York, New York.; 5 ECOG-ACRIN Biostatistical Center, Dana-Farber Cancer Institute, Boston, Massachusetts.; 6 University of Pittsburgh School of Medicine, UPMC Hillman Cancer Center, Pittsburgh, Pennsylvania.; 7 University of Pittsburgh, Pittsburgh, Pennsylvania.; 8 NRG Oncology Statistical and Data Management Center, Department of Biostatistics and Health Data Science, University of Pittsburgh, Pittsburgh, Pennsylvania.

## Abstract

Late distant recurrence (DR) remains a persistent risk in hormone receptor–positive (HR^+^) early breast cancer after completion of 5 years of endocrine therapy (ET). We developed and validated a multimodal artificial intelligence (AI) model to improve long-term risk stratification and to explore heterogeneity in benefit from extended letrozole therapy (ELT). The deep learning model integrating digitized hematoxylin and eosin whole-slide images with clinicopathologic variables was developed using 2,271 patients from the National Surgical Adjuvant Breast and Bowel Project (NSABP) B-42 trial with five-fold cross-validation and externally validated in 4,300 patients from the TAILORx trial who were disease-free at 5 years from the initial diagnosis. Prognostic performance was evaluated using hazard ratios (HR) and absolute risk differences. Exploratory analyses assessed ELT benefit across model-defined risk groups. In NSABP B-42, the model stratified patients into groups with markedly different outcomes, with a 10-year absolute DR risk difference of 7.95% between high- and low-risk groups [HR, 5.71; 95% confidence interval (CI), 3.5–9.317; *P* < 0.001]. High-risk patients derived greater absolute benefit from ELT (4.09%) than low-risk patients (0.49%). External validation in the independent TAILORx cohort confirmed prognostic performance, with MI Clarity multimodal–multitask identifying patients with significantly different late DR outcomes (HR, 1.893; 95% CI, 1.413–2.534; *P* < 0.001). This multimodal AI approach using routine pathology and clinical data enables robust and generalizable stratification of late DR risk in HR^+^ breast cancer. This scalable strategy may complement existing genomic assays and support more individualized decisions about extended ET.

**Significance:**

Late DR is a major cause of death in HR^+^ breast cancer, and deciding who needs longer hormone therapy is challenging. Current genomic tests are useful but have limitations. This study shows that an AI model using pathology and clinical data identifies patients at high or low risk of late recurrence. High-risk patients may benefit more from extended hormone therapy, offering an accessible way to guide treatment decisions.

## Introduction

Breast cancer is the most commonly diagnosed cancer and the second leading cause of cancer-related death among women in the United States (1). Approximately 70% to 80% of newly diagnosed breast cancers are hormone receptor–positive (HR^+^), which are characterized by a prolonged risk of recurrence and breast cancer mortality extending beyond 5 years of adjuvant endocrine therapy (ET; ref. 2). Extended endocrine therapy (EET) has been proposed as a strategy to mitigate this late recurrence risk, and several clinical trials have investigated its potential benefits (2–7). However, the results of these studies have been mixed, often influenced by variations in clinical endpoints and patient selection criteria. The National Surgical Adjuvant Breast and Bowel Project (NSABP) B-42 trial is a pivotal randomized, double-blind, placebo-controlled phase III study evaluating the efficacy of five additional years of letrozole after initial ET in postmenopausal women with early-stage HR^+^ breast cancer (4). The 10-year results of the trial demonstrated a statistically significant but modest benefit for extended letrozole therapy (ELT) in disease-free survival (DFS), breast cancer–free interval (BCFI), and distant recurrence (DR), with no improvement in overall survival (8).

Identifying patients most likely to benefit from EET remains a clinical challenge. Prognostic factors, including clinicopathologic features (9), circulating tumor cells (10), circulating tumor DNA (11), and molecular assays (12), have been used for late recurrence prognostication and to support EET decision-making. Breast Cancer Index (BCI), which is adopted in National Comprehensive Cancer Network (NCCN) and American Society of Clinical Oncology guidelines, consists of two components: the HOXB13/IL17BR (H/I) ratio and the molecular grade index. BCI (H/I), which is based on the H/I ratio, has demonstrated predictive capabilities for EET in multiple studies (12–14). MammaPrint (15, 16), a 70-gene assay, is relatively new in this context but has shown encouraging results in two recent studies about its potential role in identifying patients more or less likely to benefit from EET (17, 18). Both BCI and MammaPrint have been evaluated using NSABP B-42 subsets. Although BCI (H/I) showed no statistically significant treatment interaction for the primary endpoint of recurrence-free interval (RFI), time-dependent analysis of DR suggested EET benefit among BCI (H/I)-high patients beyond year four (13). Similarly, although MammaPrint showed no significant treatment interaction for the primary endpoint of DR, there was a significant treatment interaction for the secondary endpoints of DFS and BCFI, indicating more benefit for patients with low-risk MammaPrint (17).

Recent advances in computational pathology have enabled weakly supervised prediction of cancer outcomes from routine hematoxylin and eosin (H&E) whole-slide images (WSIs). Because WSIs are gigapixel-scale images with slide-level rather than tile-level outcome labels, many contemporary approaches use multiple-instance learning and attention-based aggregation to learn patient-level representations from patch-level features. These methods have demonstrated prognostic utility across breast and other cancers (19–22). In parallel, multimodal and multitask learning approaches offer a strategy to combine histologic information with clinical context and auxiliary biologically meaningful supervision, potentially improving weakly supervised representations beyond image-only prediction (23).

Leveraging these advances, we developed MI Clarity, a multimodal–multitask (M3T) deep learning framework integrating routinely available H&E WSIs with clinical variables for late DR prognostication and exploratory assessment of heterogeneity in ELT benefit in HR^+^ early breast cancer. The final M3T model combines H&E-derived image features with selected clinical variables and uses the prediction of the lowest bone mineral density (BMD) T-score as an auxiliary task during training to encourage clinically informative representations for late DR prediction. In this study, the auxiliary task was the prediction of the lowest BMD T-score. The patient’s actual T-score was used only as training-time supervision and was not provided as an input for inference. Thus, after training, model inference requires only routinely available H&E-derived image features and selected clinical variables. This design extends weakly supervised WSI prognostication to late DR risk modeling using M3T learning while avoiding dependence on the auxiliary variable at deployment. Models were developed using the NSABP B-42 translational cohort (8) and externally validated in the independent The Trial Assigning Individualized Options for Treatment (TAILORx) translational cohort (24).

## Materials and Methods

### Patient population

**Figure 1.**
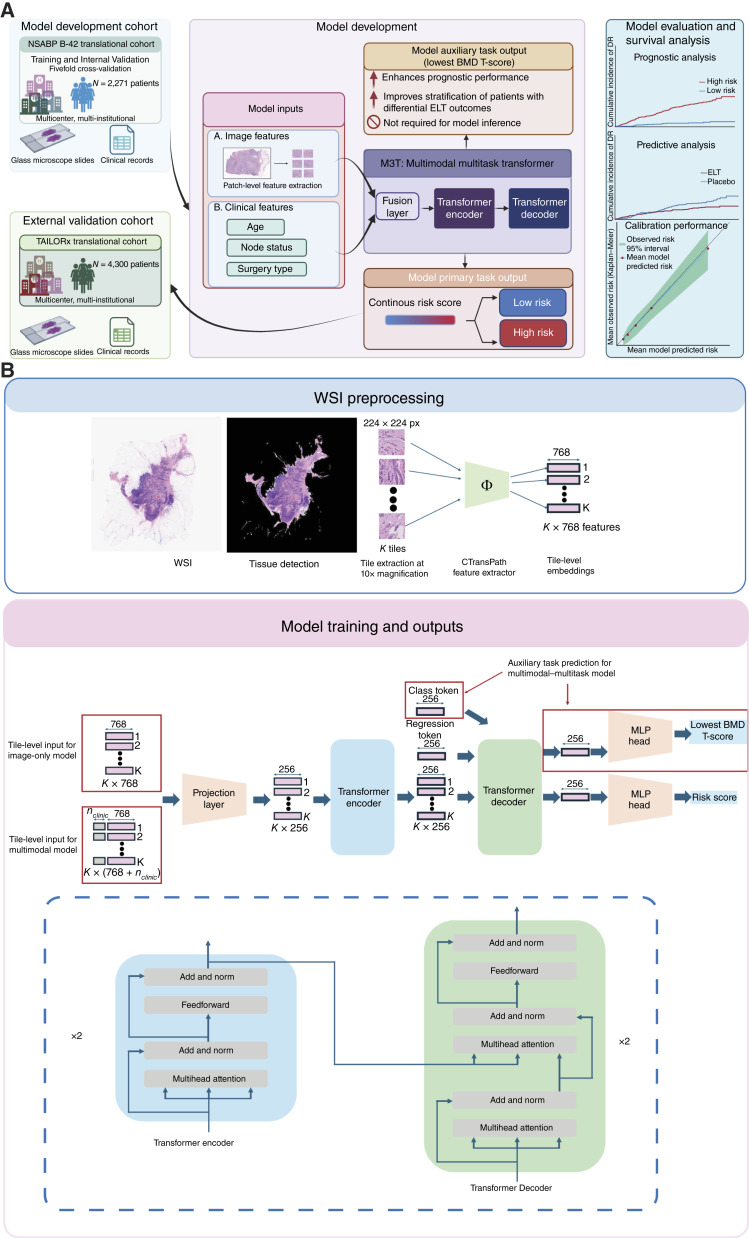
**A,** Overview of the M3T model workflow. The model was developed using the NSABP B-42 translational cohort (*N* = 2,271) with five-fold cross-validation, integrating H&E image–derived features and selected tabular clinicopathologic features through the M3T (multimodal, multitask transformer). An auxiliary task predicting the lowest BMD T-score enhanced prognostic performance and improved stratification of patients with differential ELT benefit but was not required for model inference. The model outputs a continuous risk score that is dichotomized into low- and high-risk groups. Independent external validation was performed in the TAILORx translational cohort (*N* = 4,300). Prognostic, predictive, and calibration analyses were conducted to evaluate model performance. **B,** End-to-end image preprocessing, feature extraction, model training, and output architecture. Tissue regions were detected from H&E-stained WSIs, tiled at 10× magnification, and processed using CTransPath to generate tile-level image embeddings. Tile-level image embeddings and clinicopathologic features were projected into a shared representation space and processed through transformer encoder and decoder modules. During training, an auxiliary task predicting the lowest BMD T-score was incorporated to improve prognostic performance and stratification of patients with differential ELT benefit. The primary task generated a continuous risk score for late DR risk stratification. The dashed box shows the detailed architecture of the transformer encoder (left) and transformer decoder (right).

The NSABP B-42 trial enrolled 3,966 postmenopausal women with HR^+^, early-stage breast cancer who were disease-free after 5 years of an aromatase inhibitor or tamoxifen followed by an aromatase inhibitor. Patients were randomized to receive an additional 5 years of either letrozole or placebo. A total of 2,271 eligible B-42 patients with clinical follow-up, appropriate consent, and available H&E-stained slides were included in our study (Fig. 1). For external validation, we used data from the TAILORx trial. Overall DR prognostication was evaluated among 6,516 patients with available H&E slides and relevant clinical features (age at TAILORx randomization, pathologic node status, surgery type) for model risk score generation. WSIs were processed using the same workflow as the B-42 cohort, and MI Clarity risk scores and categorical risk labels (high vs. low) were derived. To approximate the B-42 design, age at TAILORx randomization plus 5 years was used to reflect age after completion of initial ET. Late DR prognostication was evaluated among 4,300 patients who had completed ≥4.5 years of ET and were disease-free at the 5-year landmark (Fig. 1).

This study was conducted in accordance with the guidelines of the Declaration of Helsinki, the Belmont Report, and the U.S. Common Rule. This study used retrospective, deidentified data and was approved with a waiver of written informed consent (21 CFR 50.22) by the WCG Institutional Review Board.

### Model development overview

We developed a multimodal deep learning framework (MI Clarity) using digitized H&E slides from the NSABP B-42 translational cohort. Three model variants were evaluated: an image-only model, a multimodal model integrating histopathology and clinical variables, and a M3T model incorporating an auxiliary task to enhance feature learning (Fig. 1).

Briefly, WSIs were converted to TIFF format, tissue regions were identified using a QuPath-based pixel classifier, and artifacts such as coverslip edges and pen markings were excluded using HistoQC (RRID:SCR_025780; ref. 25). Tissue regions were tiled at 10× magnification into 224 × 224-pixel patches, and a pretrained CTransPath (26) model was used to generate 768-dimensional tile-level embeddings. For transformer-based risk prediction, tile embeddings were processed using an encoder–decoder architecture to generate a whole-slide risk score. In the multimodal and M3T models, selected clinical variables, including age at NSABP B-42 randomization, pathologic node status, and surgery type, were integrated with image-derived features using early fusion. The M3T model additionally incorporated a training-time auxiliary task predicting the lowest BMD T-score status to improve representation learning; this auxiliary variable was not required for model inference. Additional details on image preprocessing, feature extraction, model architecture, cross-validation, model training, risk-score threshold selection, and locked external validation in TAILORx are provided in the Supplementary Methods.

### Statistical analysis

#### Prognostic, predictive, and calibration analysis in the NSABP B-42 translational cohort

Clinical representativeness of the translational cohort was assessed using *χ*^2^ tests. The primary endpoint for model evaluation was DR. DR was defined as the time from NSABP B-42 randomization to the first documented DR; patients without DR were censored at the date of the last clinical follow-up. Because B-42 randomization occurred after the completion of approximately 5 years of prior ET, model evaluation focused on late DR risk in the EET setting. No formal prospective *a priori* power calculation was performed because this was a retrospective analysis of available H&E-stained slides and clinical outcomes from the completed B-42 trial. The evaluable sample size was determined by tissue availability, slide quality, consent, clinical follow-up, and observed DR events. Prognostic performance was evaluated using Kaplan–Meier estimates, log-rank tests, Cox models, and 10-year absolute risk differences. Multivariable Cox models for DR included model-predicted risk groups, treatment arms, and prespecified clinical covariates. Proportional hazards assumptions were examined, and time-dependent or stratified Cox models were applied when necessary. For predictive analyses of ELT benefit, stratified log-rank tests, stratified Cox models, and interaction terms between treatment and risk group were evaluated. Model calibration was evaluated at prespecified time horizons to assess agreement between predicted and observed risks.

#### External validation in the TAILORx translational cohort

For external validation, the primary endpoint was DR-free interval (DRFI). Prognostic performance was assessed using Kaplan–Meier analysis, log-rank tests, and Cox models. Multivariable Cox models were adjusted for age, menopausal status, surgery type, chemotherapy receipt, tumor size, tumor grade, and the Oncotype DX Recurrence Score. All analyses were performed both in the full DR cohort (*n* = 6,516) and in the late-DR cohort (*n* = 4,300).

#### Model interpretation analysis

To evaluate the histopathologic patterns learned by MI Clarity, we performed model interpretation analyses using tile-level embeddings and WSI-level heatmaps. For spatial visualization, attention, patch-level risk, and contribution heatmaps were generated by mapping tile-level model outputs back to their original WSI coordinates. Attention scores were derived from self-attention values in the second decoder block and represented regions prioritized by the model during slide-level prediction. Patch-level risk scores were estimated by passing each tile individually through the network. Contribution scores were calculated as the product of the single-tile risk score and the corresponding attention score, thereby identifying regions with the greatest influence on the final WSI-level prediction.

To further characterize morphologic patterns associated with model-predicted risk, we selected 20 representative WSIs, including the 10 slides with the highest image-only predicted risk scores and the 10 slides with the lowest image-only model-predicted risk scores. We focused on the image-only model for tile-level morphologic interpretation so that patch-level risk scores reflected image-derived features alone, without contribution from clinical variables or auxiliary multitask supervision. Tile-level CTransPath (26) embeddings were clustered using K-means and visualized using Uniform Manifold Approximation and Projection for Dimension Reduction (UMAP; RRID:SCR_018217). Patch-level risk scores were compared across clusters, and representative tiles were reviewed to characterize dominant morphologic features. For representative use cases comparing image-only and multimodal prediction, heatmaps were generated separately using the image-only and M3T models. Additional details are provided in the Supplementary Methods.

## Results

### Patient characteristics of the NSABP B-42 translational cohort

Of 3,966 randomized patients, 2,271 (57.3%) from the NSABP B-42 trial with available clinical outcomes and digitized H&E slides were included. This translational cohort was similar to the remaining B-42 population in clinicopathologic and treatment features, with minor differences in pathologic node status and HER2 status (Supplementary Table S1).

### Prognostic performance of MI Clarity in the NSABP B-42 translational cohort

The image-only model, based solely on histopathologic features from WSIs, demonstrated strong prognostic performance for late DR. Using a predefined risk score threshold derived from the training dataset, high-risk patients (risk score ≥ threshold) had significantly worse outcomes than low-risk patients [hazard ratio (HR) = 3.419; 95% confidence interval (CI), 2.241–5.215; *P* < 0.001] and a 10-year absolute difference in DR of 5.79%. Similar trends were observed among patients receiving ELT (HR = 3.206; 95% CI, 1.664–6.174; *P* < 0.001) or placebo (HR = 3.571; 95% CI, 2.053–6.211; *P* < 0.001; Supplementary Table S2). This association remained significant in multivariable analysis (HR = 2.761; 95% CI, 1.799–4.238; *P* < 0.001; Supplementary Table S3).

**Figure 2.**
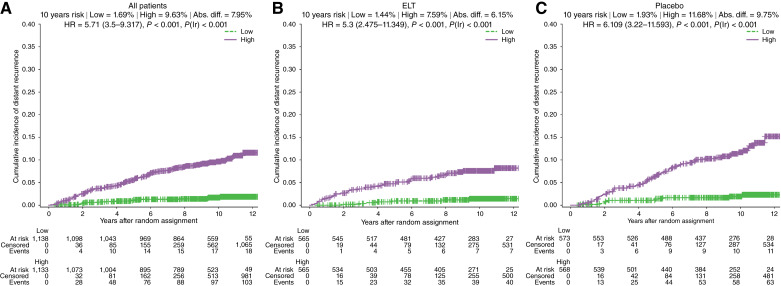
Kaplan–Meier analysis of DR in the NSABP B-42 translational cohort according to MI Clarity M3T risk groups. **A,** All patients. **B,** ELT-treated group. **C,** Placebo group. High- and low-risk groups are defined by the M3T model.

Adding clinical variables improved model performance, with the multimodal model yielding an HR of 4.507 (95% CI, 2.863–7.096; *P* < 0.001; Supplementary Table S2). The M3T model further improved stratification (HR = 5.710; 95% CI, 3.500–9.317; *P* < 0.001), increasing the absolute DR difference to 7.95% (Fig. 2A). Model performance was consistent across ELT and placebo arms (Fig. 2B and C). We further performed the same multivariable Cox analysis for the M3T model. The M3T risk group remained independently associated with DR; patients classified as high-risk by the M3T model had a significantly higher risk of DR than those classified as low-risk (adjusted HR = 3.575; 95% CI, 2.039–6.269; *P* < 0.001; Supplementary Table S4). Worse outcomes were also observed for RFI, BCFI, and DFS among high-risk patients (Supplementary Table S5). Baseline clinicopathologic and treatment characteristics of patients included in the M3T model analysis are shown in Supplementary Table S6.

### Calibration of 10-year distant recurrence risk

At the 10-year horizon, all three models demonstrated acceptable overall calibration, with mean predicted risks closely aligned with the observed event rate (5.5%) and calibration-in-the-large values near zero, indicating minimal global bias. Among the three models, the M3T model showed the most favorable calibration profile, with the lowest expected calibration error and the smallest median maximum bin error (0.021), indicating improved local calibration (Supplementary Table S7). Calibration slopes for all models were close to 1, reflecting appropriate risk scaling across the risk range, with comparable variability across repeated splits (Supplementary Fig. S1). Overall, calibration performance was acceptable for all models, with the M3T model providing the most stable bin-level agreement between predicted and observed risks.

### Model interpretation identifies recurrence-associated morphologic patterns

**Figure 3.**
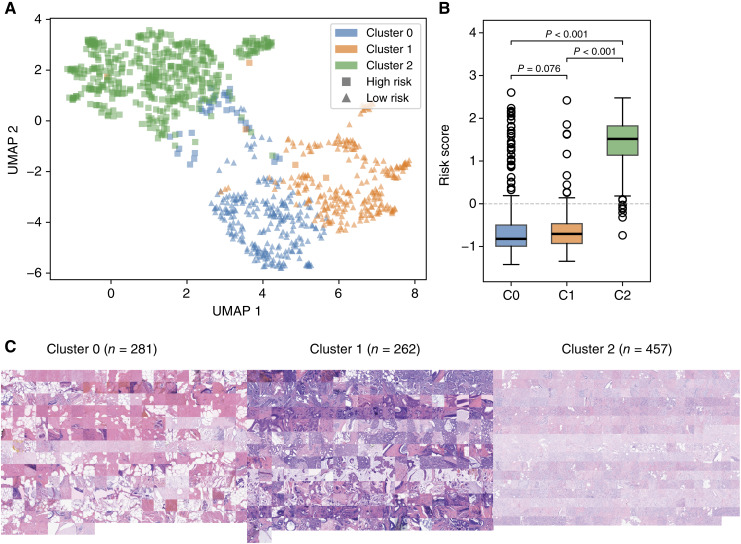
Model interpretation using tile-level embeddings and whole-slide spatial heatmaps. **A,** UMAP visualization of tile-level histopathology embeddings extracted from H&E-stained WSIs. Points are colored by unsupervised embedding cluster and shaped according to model-defined risk group. **B,** Distribution of model-predicted risk scores across embedding clusters. Boxplots indicate the median and interquartile range, with pairwise comparison *P* values shown. **C,** Representative image tiles from each embedding cluster, illustrating morphologic patterns captured by the image feature extractor.

We performed a tile-level analysis to link learned histopathology features with patch-level risk scores. Tile-level embeddings separated into three morphologically coherent clusters on UMAP visualization (Fig. 3A–C). Patch-level risk scores differed significantly across clusters. Cluster 2 had significantly higher patch-level risk scores than clusters 0 and 1, whereas clusters 0 and 1 were not significantly different at the conventional threshold. Cluster 0 was dominated by low-cellularity, fibroblast-rich, and collagen-rich background with low tumor content. Cluster 1 was enriched for epithelial-rich morphology, including crowded epithelial nests, ductal irregular glands, focal solid epithelial groups, luminal structures, and tumor-like epithelial fragments. Cluster 2 was enriched for invasive carcinoma–like morphology with desmoplastic stroma, including infiltrative glands, nests, cords, and tubules embedded within reactive collagenous stroma. Together, these findings linked the highest-risk image-feature cluster to an invasive carcinoma–like/desmoplastic tumor–stroma phenotype.

To visualize these signals spatially, we generated WSI-level heatmaps of model attention score, patch-level risk score, and attention-weighted contribution score. Attention maps identified regions prioritized by the model, patch-level risk maps showed the direction and magnitude of local recurrence–associated signals, and contribution maps integrated attention and risk to identify regions with the greatest influence on slide-level prediction. In representative use cases, heatmaps localized high-risk and high-contribution regions predominantly within tumor-bearing tissue. In a discordant case, the image-only model classified the patient as low-risk, whereas the M3T model classified the patient as high risk, consistent with observed DR. In another case from the clinically low-risk subgroup, M3T identified high-risk regions within tumor-bearing tissue despite favorable clinicopathologic features (Supplementary Fig. S2). These analyses support the biological plausibility of MI Clarity and illustrate how multimodal integration can refine recurrence-risk prediction beyond image-only or clinical assessment alone.

### Exploratory assessment of ELT benefit by MI Clarity–defined risk groups

**Figure 4.**
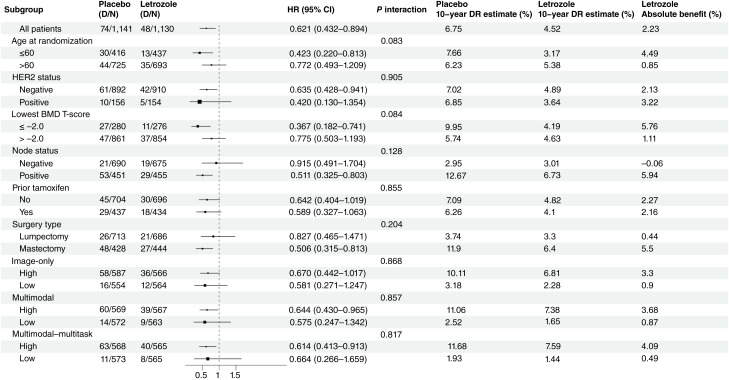
Effect of ELT on DR across subgroups in the NSABP B-42 translational cohort.

Beyond stratifying prognosis, MI Clarity may also support treatment decision-making for ELT. In the overall translational cohort, ELT lowered the 10-year risk of DR by 2.23% (HR = 0.621; 95% CI, 0.432–0.894; *P* = 0.01; Supplementary Table S8). Subgroup analyses revealed larger absolute benefits among patients who were node-positive, had undergone mastectomy, were aged ≤60 years, had HER2^+^ disease, presented with a lowest BMD T-score ≤ −2, or were classified as high-risk by the image-only, multimodal, or M3T model variants (Fig. 4). For example, ELT conferred virtually no benefit in node-negative patients (−0.06%), whereas node-positive patients experienced a 5.94% benefit and a significant risk reduction (HR = 0.511; 95% CI, 0.325–0.803; *P* = 0.003). Patients treated by mastectomy, a procedure typically reserved for larger tumors, gained a 5.5% absolute benefit, compared with 0.44% in lumpectomy recipients (Fig. 4; Supplementary Table S9).

**Figure 5.**
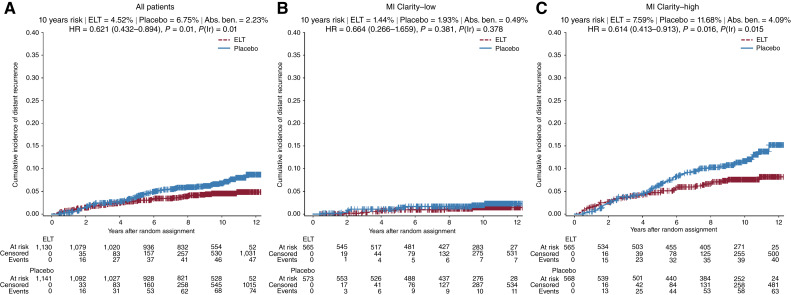
Kaplan–Meier analysis of DR comparing patients treated with ELT vs. placebo in the NSABP B-42 translational cohort. **A,** All patients. **B,** Low-risk group. **C,** High-risk group. High- and low-risk groups are defined by the M3T model.

Risk stratification by the image-only model indicated a 3.3% absolute ELT benefit in the high-risk group (HR = 0.67; 95% CI, 0.442–1.017; *P* = 0.058) and 0.9% in the low-risk group (HR = 0.581; 95% CI, 0.271–1.247; *P* = 0.159; Supplementary Table S8). The multimodal and M3T models refined this separation. For the M3T model, high-risk patients derived a 4.09% absolute benefit (HR = 0.614; 95% CI, 0.413–0.913; *P* = 0.015), whereas low-risk patients accrued only a 0.49% absolute benefit (HR = 0.664; 95% CI, 0.266–1.659; *P* = 0.378; Fig. 5; Supplementary Table S8). Interaction terms between treatment and risk group were not significant (*P* = 0.868 for the image-only and 0.817 for the M3T model), yet the magnitude of absolute benefit consistently favored high-risk patients (Supplementary Table S8). Subgroup analyses using the M3T model further supported these patterns (Supplementary Tables S9 and S10). In mastectomy patients, a significant treatment-by-risk interaction was observed. In node-positive patients (*n* = 906), high-risk individuals (81.4%) experienced a 6.83% absolute benefit, whereas low-risk patients (18.6%) showed only 1.5% (Supplementary Fig. S3).

### External validation of MI Clarity prognostic performance in the TAILORx translational cohort

**Figure 6.**
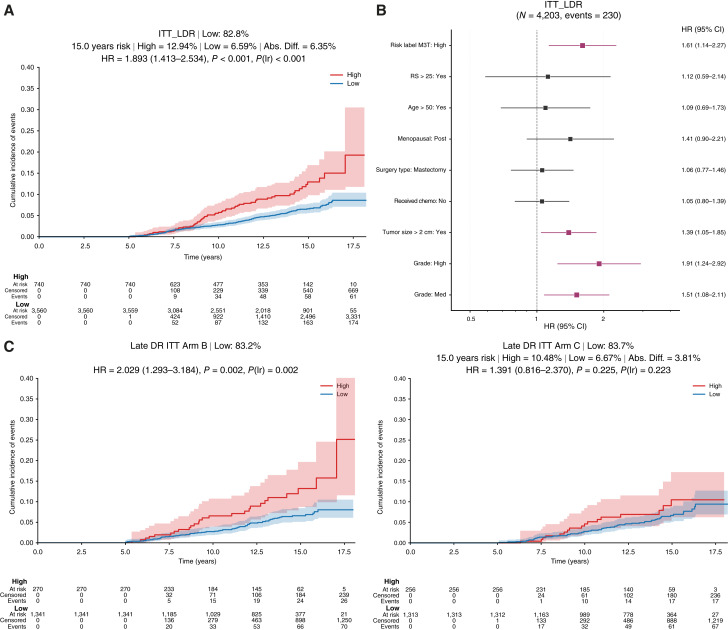
External validation of MI Clarity M3T for late DR in the TAILORx translational cohort. **A,** Kaplan–Meier analysis of late DR comparing MI Clarity M3T risk groups in all patients. **B,** Multivariable Cox analysis assessing the independent prognostic value of the MI Clarity M3T risk label, adjusted for clinical covariates. **C,** Kaplan–Meier analysis of late DR comparing MI Clarity M3T risk groups in arms B and C (ITT). LDR, late distant recurrence.

Among the 4,300 patients in the late-DR TAILORx cohort, 3,560 (82.8%) were classified as low-risk and 740 (17.2%) as high-risk by the MI Clarity M3T model (Supplementary Table S11). High-risk patients had significantly worse outcomes than low-risk patients (HR = 1.893; 95% CI, 1.413–2.534; *P* < 0.001), with a 15-year absolute DR risk difference of 6.35% (Fig. 6A). In multivariable Cox analysis, the MI Clarity M3T-derived risk label remained an independent prognostic factor (adjusted HR = 1.61; 95% CI, 1.14–2.27; *P* = 0.007; Fig. 6B).

In the Oncotype DX intermediate-risk group (RS 11–25), patients randomized to ET (arm B) or chemoendocrine therapy (arm C) were evaluated using intention-to-treat (ITT), as-treated, and per-protocol analyses. In the ITT population, MI Clarity M3T risk stratification was highly prognostic in arm B (HR = 2.029; 95% CI, 1.293–3.184; *P* = 0.002), whereas the association was not significant in arm C (Fig. 6C).

We further evaluated overall DR prognostication among the entire cohort of 6,516 patients at initial diagnosis and enrollment in TAILORx, of whom 1,134 (17.4%) were classified as high-risk and 5,382 (82.6%) as low-risk. Across the entire follow-up period from TAILORx randomization, high-risk patients continued to experience significantly worse outcomes (HR = 1.734; 95% CI, 1.398–2.152; *P* < 0.001), with a 15-year absolute DR risk difference of 7.09%. Multivariable analysis (*n* = 6,354) confirmed M3T’s independent prognostic value in this cohort (adjusted HR = 1.36; 95% CI, 1.05–1.77; *P* = 0.02; Supplementary Fig. S4).

## Discussion

Building on recent advancements in pathology foundation models and advanced algorithms (26–29), we developed MI Clarity, an artificial intelligence system that combines unannotated H&E-stained slides and clinical data to provide long-term DR prognostication and to identify patients most likely to benefit from EET. In the NSABP B-42 translational cohort, all model variants demonstrated strong prognostic performance and identified a subgroup of high-risk patients with a significant benefit from ELT, whereas no significant benefit was observed in the low-risk group. Among these models, the M3T model, which integrates H&E images and selected baseline clinical features within a multitask framework and incorporates an auxiliary lowest BMD T-score prediction task, demonstrated the greatest risk discrimination. In addition, the M3T model showed the largest absolute ELT benefit among high-risk patients in the NSABP B-42 translational cohort. Although the interaction *P* value for treatment effect was not significant in the overall cohort, a significant interaction was observed in the mastectomy subgroup, suggesting potential predictive value in this subset.

MI Clarity builds on established weakly supervised computational pathology approaches, including multiple-instance learning and attention-based aggregation of patch-level image features. The novelty of the M3T framework is not the use of attention-based WSI aggregation alone but rather its integration of image-derived and clinicopathologic features with training-time auxiliary supervision for late DR prediction. The auxiliary BMD T-score task was used only during training to encourage clinically informative representation learning and was not required for inference. The observed improvement from the image-only model to the multimodal and M3T models supports the contribution of multimodal integration and auxiliary multitask learning beyond a standard image-only framework.

Model interpretation analyses further support the biological plausibility of MI Clarity and help address concerns about black box prediction. Tile-level embedding analysis identified morphologically coherent clusters, with the highest-risk cluster enriched for invasive carcinoma–like morphology with desmoplastic stroma, including infiltrative glands, nests, cords, and tubules embedded in reactive collagenous stroma. Spatial heatmaps localized high-risk and high-contribution regions predominantly to tumor-bearing tissue. These findings suggest that MI Clarity’s recurrence-risk signal is at least partly grounded in recognizable tumor–stroma morphology rather than nonspecific slide-level artifacts.

To further assess the clinical reliability of DR risk predictions, we evaluated model calibration at clinically relevant time horizons. At the 10-year horizon, all models showed acceptable overall calibration, with mean predicted risks closely matching the observed event rate and minimal global bias. The M3T model demonstrated the most favorable calibration profile, suggesting that M3T learning may also support more stable risk estimation.

We compared MI Clarity with BCI, previously evaluated in a separate cohort from the NSABP B-42 trial. In the BCI analysis (13), the treatment × BCI (H/I) interaction was not significant for DR (*P*_interaction_ = 0.71). The absolute 10-year DR benefit was 1.4% for BCI (H/I)-low (HR = 0.59; 95% CI, 0.33–1.03; *P* = 0.06) and 3.1% for BCI (H/I)-high patients (HR = 0.68; 95% CI, 0.42–1.12; *P* = 0.13). In our study, the MI Clarity M3T model identified high-risk patients with a 4.09% absolute benefit and low-risk patients with an estimated 10-year DR risk below 2% in both the ELT (1.44%) and placebo (1.93%) arms (absolute benefit of ELT: 0.49%; Supplementary Table S12). These findings suggest that MI Clarity provides prognostic and exploratory predictive information comparable with established genomic assays such as BCI when evaluated within subsets of the NSABP B-42 trial.

Subgroup analyses in the NSABP B-42 translational cohort demonstrated the model’s ability to refine decision-making beyond traditional clinicopathologic features. For example, in mastectomy patients, MI Clarity M3T identified a significant treatment-by-risk interaction: high-risk patients derived an 8.69% absolute benefit from ELT, whereas low-risk patients experienced a −2.67% absolute benefit. In node-positive patients, typically considered high-risk, the M3T model identified a distinct low-risk subset (18.6%) deriving minimal ELT benefit (1.5%), in contrast to a 6.83% benefit among high-risk patients. These trends support the ability of MI Clarity to personalize treatment—minimizing overtreatment while targeting those most likely to benefit.

To evaluate generalizability, we externally validated MI Clarity M3T in the independent TAILORx translational cohort. In this cohort, high-risk patients experienced significantly worse late DR outcomes. Across ITT, as-treated, and per-protocol analyses, risk separation was stronger among patients in arm B than in arm C, suggesting that chemotherapy exposure may partially attenuate observed prognostic differences. Importantly, beyond late events, MI Clarity M3T also provided significant prognostic information for overall DR across all 6,516 patients. Together, these findings demonstrate consistent and clinically meaningful prognostic performance of MI Clarity M3T across analytic frameworks and endpoints.

Several contextual considerations should be acknowledged when interpreting these results. First, as detailed in the Supplementary Methods, endpoint definitions differed slightly between trials. In B-42, DR was assessed from trial randomization (which was after patients had completed about 5 years of adjuvant ET), thereby restricting analyses to patients who remained disease-free at that landmark. In contrast, TAILORx evaluated DRFI from initial randomization (which occurred before any adjuvant systemic therapy) and classified deaths as events when DR was the first manifestation. To enable cross-trial comparison, we approximated a late DR population in TAILORx by including patients who had completed ≥4.5 years of ET and were disease-free at the 5-year landmark. Although TAILORx included only node-negative patients, its late-DR validation cohort had a higher observed event rate than the node-negative subgroup of B-42 [235/4,300 (5.5%) vs. 40/1,365 (2.9%)]. This higher baseline event burden provides an important context for interpreting the less favorable absolute outcomes in the TAILORx low-risk group. Second, chemotherapy exposure was not captured in B-42, limiting the assessment of treatment effect heterogeneity in that trial. Third, although MI Clarity was validated in two large clinical trials with long-term follow-up, predictive validation for EET remains incomplete because TAILORx was not designed for this purpose; in fact, approximately 85.7% of the 4,300 patients included in the TAILORx late-DR validation cohort received some EET beyond 5 years, potentially attenuating prognostic discrimination. These differences reflect trial-specific design features but do not detract from the overall consistency of MI Clarity’s prognostic performance across studies.

Beyond these design-specific differences, notable cohort characteristics should also be considered. The B-42 trial enrolled both node-positive and node-negative patients, whereas TAILORx included only node-negative disease. B-42 restricted eligibility to postmenopausal women, whereas TAILORx enrolled both pre/peri- and postmenopausal patients. These clinical differences may partially explain the higher proportion of patients classified as low-risk in TAILORx compared with B-42. In B-42, adding clinicopathologic covariates to the M3T model reclassified node-negative patients from 56.4% to 71% in the low-risk group and node-positive patients from 61.5% to 81.3% in the high-risk group, compared with the image-only model (Supplementary Table S13). Consistently, in TAILORx (all node-negative), 82.8% of patients were classified as low-risk by the M3T model. These findings show that integrating clinical factors improves model calibration and produces risk assignments that better reflect established clinical patterns.

Together, these findings highlight the importance of accounting for baseline clinical differences across trials when evaluating model generalizability. Despite these distinctions, MI Clarity maintained robust prognostic performance across diverse trial designs, patient populations, and analytic approaches.

Finally, both NSABP B-42 and TAILORx predated the contemporary use of adjuvant CDK4/6 inhibitors in selected moderate- to high-risk HR^+^ early breast cancer (30, 31). Therefore, absolute recurrence risks observed in these cohorts may differ from those expected in contemporary patients receiving modern systemic therapy. Although this study was designed to evaluate late DR risk and exploratory heterogeneity in ELT benefit rather than CDK4/6 inhibitor treatment selection, future validation in contemporary cohorts will be important to define the performance of MI Clarity in the modern treatment era.

In addition to its generalizability across cohorts, MI Clarity offers several practical advantages over molecular assays. Genomic profiling tests such as Oncotype DX (32), BCI (33), and MammaPrint (15) have been widely adopted for DR prognostication and treatment guidance. Although these assays are incorporated into NCCN guidelines to help identify patients who might benefit from chemotherapy or EET, their high-cost and time-intensive nature may limit accessibility. In contrast, MI Clarity offers a potentially cost-effective and scalable alternative by leveraging routinely available H&E-stained slides and clinical features to provide prognostic information within hours. MI Clarity may also serve as an orthogonal tool alongside genomic tests to strengthen decision-making. By leveraging routine histology, it has the potential to reduce overtreatment, improve patient outcomes, and broaden access to precision prognostication.

## Supplementary Material

Supplementary Figure 1 Calibration plots showing observed versus predicted 10-year distant recurrence risk.

Supplementary Figure 2 Representative cases.

Supplementary Figure 3 Kaplan-Meier analysis of distant recurrence comparing patients treated with ELT versus placebo in subsets of patients from NSABP B-42 translational cohort defined by pathological node status.

Supplementary Figure 4 External validation of MI Clarity M3T for overall distant recurrence (DR) in the TAILORx translational cohort.

Supplementary Methods Supplementary Methods

Supplementary Table 1 Clinicopathologic and treatment characteristics of patients in the translational cohort versus remaining NSABP B-42 patients.

Supplementary Table 2 Prognostication performance comparison of models (image-only, multimodal, M3T model) for risk of distant recurrence (DR).

Supplementary Table 3 Results of the multivariable analysis of DR for the image-only model.

Supplementary Table 4 Results of the multivariable analysis of DR for the M3T model.

Supplementary Table 5 Prognostic performance of MI Clarity M3T model for breast cancer-free interval (BCFI), disease-free survival (DFS) and recurrence-free interval (RFI).

Supplementary Table 6 Clinicopathologic, and treatment characteristics of patients classified as MI Clarity–High versus Low risk in the NSABP B-42 translational cohort.

Supplementary Table 7 Calibration performance of models for 10-year distant recurrence risk in the NSABP B-42 translational cohort.

Supplementary Table 8 Performance comparison of models (image-only, multimodal, M3T model) for predicting benefit from ELT.

Supplementary Table 9 Predictive performance of the MI Clarity M3T model for ELT benefit across clinical subgroups.

Supplementary Table 10 Time-dependent predictive performance of the M3T model for ELT benefit in clinical subgroups with violated proportional hazards (PH) assumption.

Supplementary Table 11 Clinicopathologic, and treatment characteristics of patients classified as MI Clarity–High versus Low risk in the TAILORx translational late-DR cohort.

Supplementary Table 12 Predictive performance comparison of Clarity models with Breast Cancer Index (BCI).

Supplementary Table 13 Distribution of model predicted risk groups by pathological node status across different models.

Supplementary Code MI Clarity code

## Data Availability

The raw WSIs and patient-level clinical and outcome data used in this study are derived from the NSABP B-42 (4, 8) and TAILORx (24) clinical trial translational cohorts and are subject to trial governance, participant consent, patient privacy protections, and data-use restrictions. Therefore, the raw data used in this study cannot be made publicly available. Qualified investigators may request access to the relevant trial data through the appropriate cooperative group or trial data-access mechanisms, subject to approval and execution of applicable data-use agreements (visit https://www.nrgoncology.org/clinical-trials/clinical-trial-resources/biospecimens-access/ to request access to B-42 data and https://dbgap.ncbi.nlm.nih.gov/beta/study/phs004260.v1.p1/#study for TAILORx data).

## Code Availability

The code associated with MI Clarity is included within the Supplementary Materials.
