# Supplementary material for: Development and Validation of a Multimodal–Multitask Deep Learning Approach for Estimating Late Distant Recurrence Risk in HR-Positive Early Breast Cancer
Source: Cancer Res Commun. 2026 Jul 31;6(7):1825–35. doi: 10.1158/2767-9764.CRC-26-0362 (PMC13425195; doi:10.1158/2767-9764.CRC-26-0362)

**Supplementary Figure 1. Calibration plots showing observed versus predicted 10-year distant recurrence risk. A.** image-only **B.** multimodal **C.** multimodal-multitask (M3T)


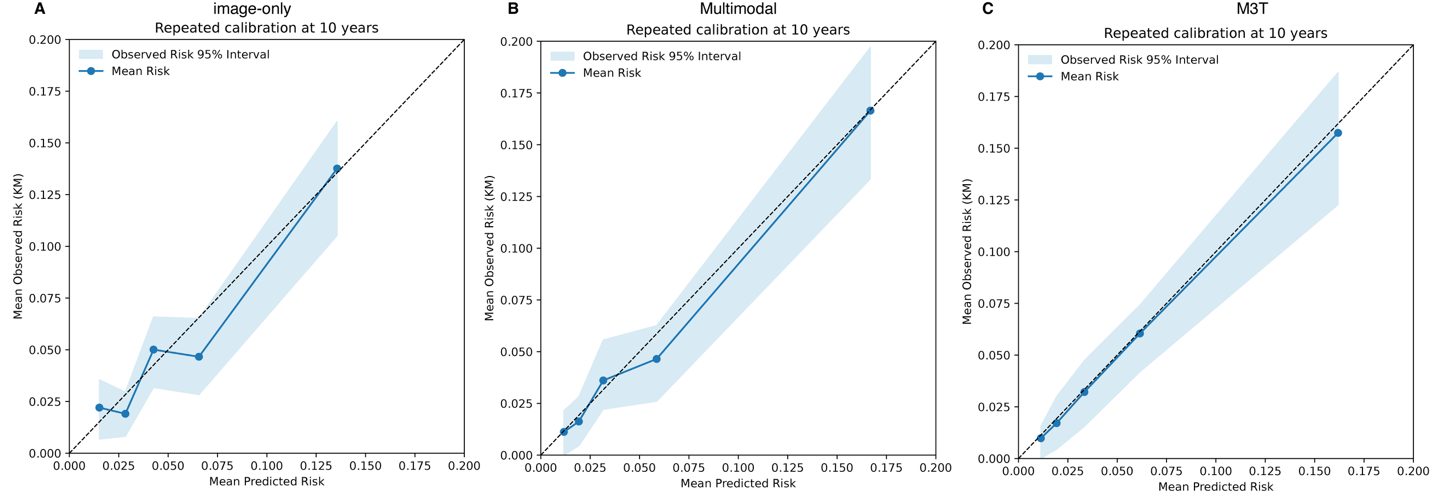

Supplement: Supplementary Figure 1 — Calibration plots showing observed versus predicted 10-year distant recurrence risk. [file crc-26-0362_supplementary_figure_1_suppsf1.docx]
