# Supplementary material for: Development and Validation of a Multimodal–Multitask Deep Learning Approach for Estimating Late Distant Recurrence Risk in HR-Positive Early Breast Cancer
Source: Cancer Res Commun. 2026 Jul 31;6(7):1825–35. doi: 10.1158/2767-9764.CRC-26-0362 (PMC13425195; doi:10.1158/2767-9764.CRC-26-0362)

**Supplementary Figure 2.** **Representative cases.** **A.** Representative discordant case comparing the image-only model with the multimodal M3T model. The image-only model classified the patient as low risk, whereas the M3T model classified the patient as high risk, consistent with the observed distant recurrence. For each model, the original H&E-stained whole-slide image is shown with corresponding heatmaps of model attention score, patch-level risk score, and attention-weighted contribution score. **B.** Representative case from the clinically low-risk subgroup. Despite favorable clinical features, including node-negative disease and lumpectomy, the patient developed distant recurrence and was classified as high risk by the M3T model. Spatial heatmaps show that high-risk and high-contribution regions were localized predominantly within tumor-bearing tissue.


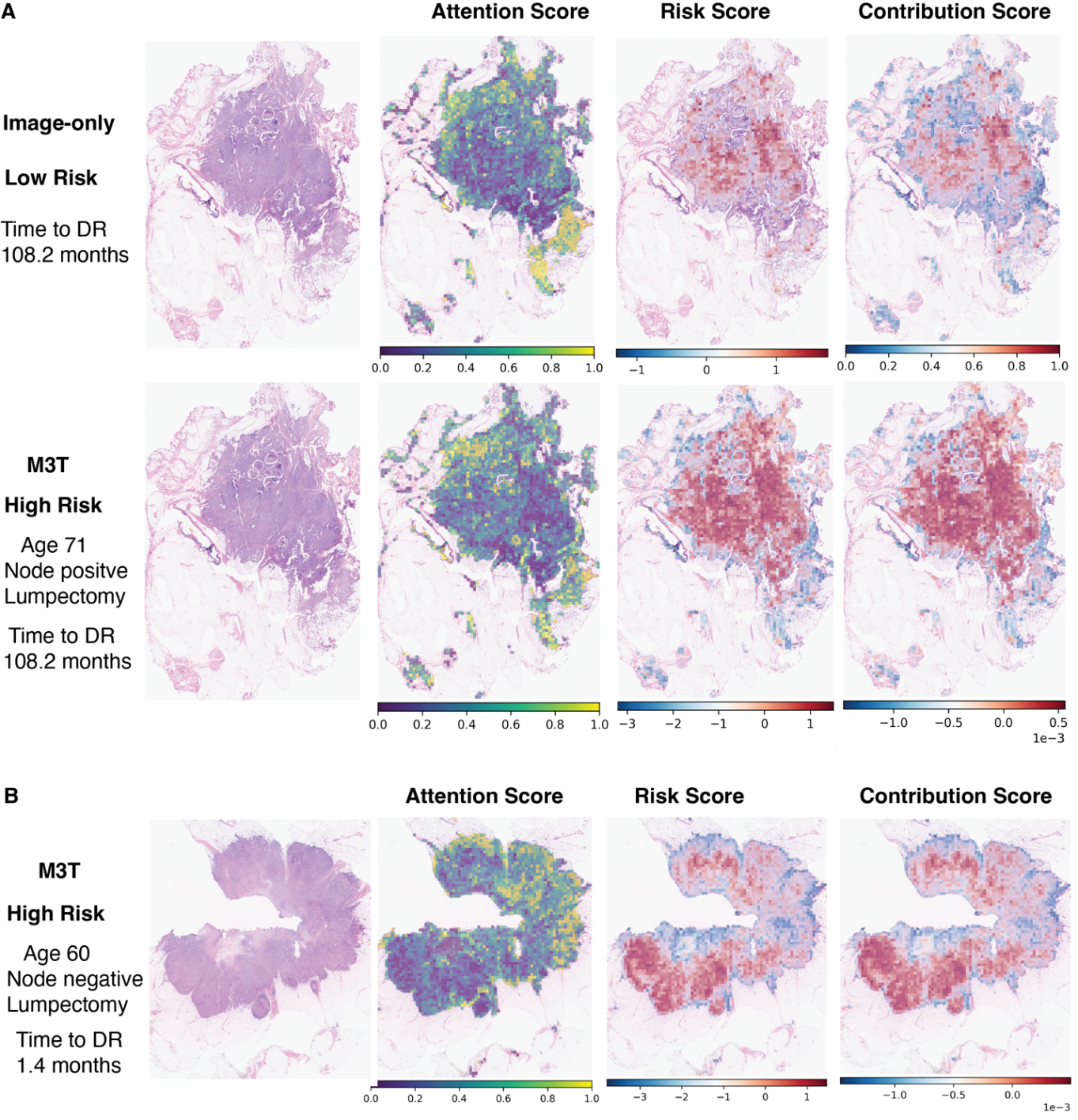

Supplement: Supplementary Figure 2 — Representative cases. [file crc-26-0362_supplementary_figure_2_suppsf2.docx]
