# Supplementary material for: Development and Validation of a Multimodal–Multitask Deep Learning Approach for Estimating Late Distant Recurrence Risk in HR-Positive Early Breast Cancer
Source: Cancer Res Commun. 2026 Jul 31;6(7):1825–35. doi: 10.1158/2767-9764.CRC-26-0362 (PMC13425195; doi:10.1158/2767-9764.CRC-26-0362)

**Supplementary Figure 3. Kaplan-Meier analysis of distant recurrence comparing patients treated with ELT versus placebo in subsets of patients from NSABP B-42 translational cohort defined by pathological node status. A.** Node-negative (N0). **B.** Node-positive (N+), **C.** Time-dependent analysis of N+ patients in the overall translational cohort and according to MI Clarity multimodal-multitask (M3T) model.


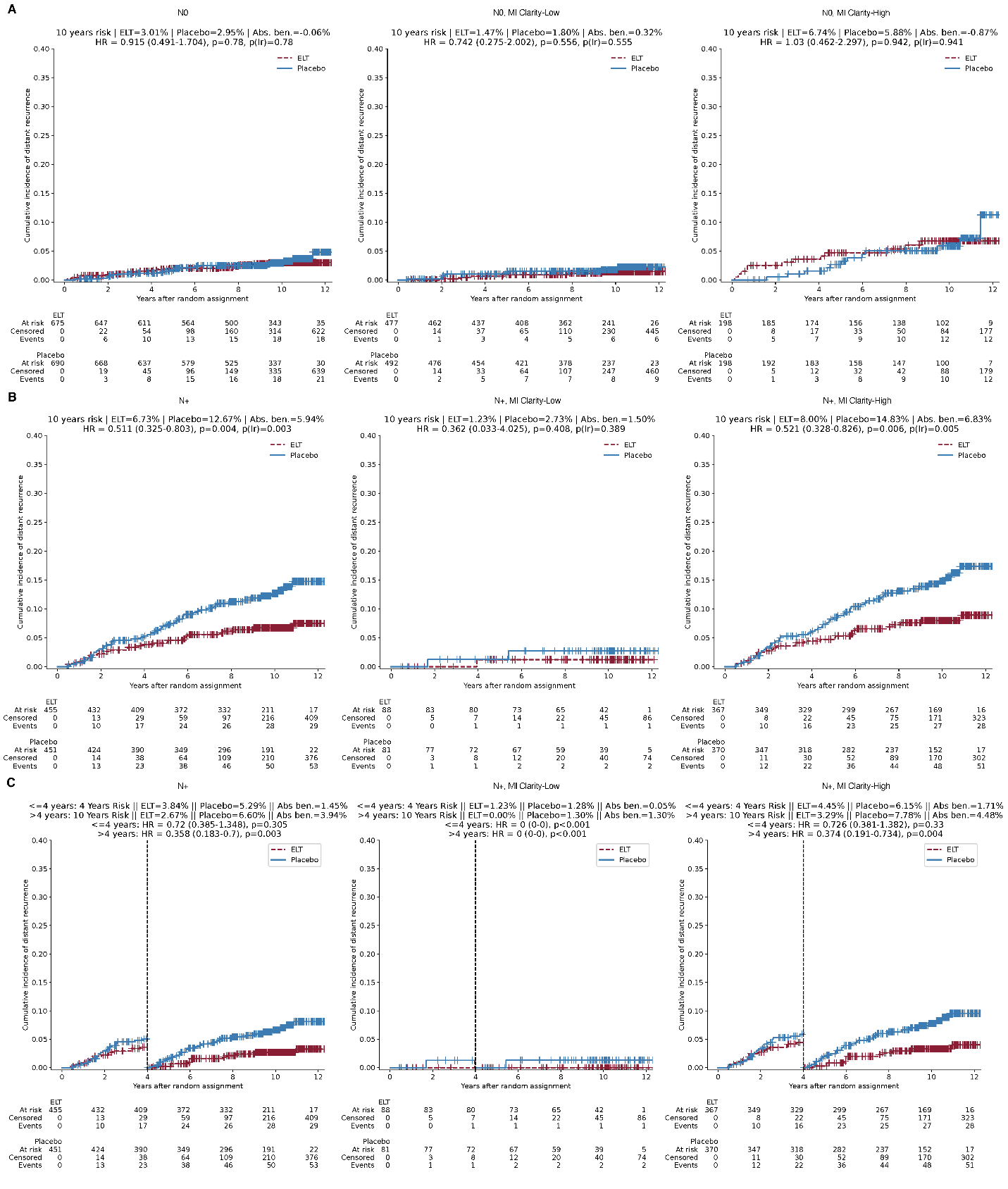

Supplement: Supplementary Figure 3 — Kaplan-Meier analysis of distant recurrence comparing patients treated with ELT versus placebo in subsets of patients from NSABP B-42 translational cohort defined by pathological node status. [file crc-26-0362_supplementary_figure_3_suppsf3.docx]
