# Supplementary material for: Development and Validation of a Multimodal–Multitask Deep Learning Approach for Estimating Late Distant Recurrence Risk in HR-Positive Early Breast Cancer
Source: Cancer Res Commun. 2026 Jul 31;6(7):1825–35. doi: 10.1158/2767-9764.CRC-26-0362 (PMC13425195; doi:10.1158/2767-9764.CRC-26-0362)

**Supplementary Figure 4. External validation of MI Clarity M3T for overall distant recurrence (DR) in the TAILORx translational cohort. A.** Kaplan–Meier analysis of overall DR comparing MI Clarity M3T risk groups in all patients. **B.** Multivariable Cox analysis assessing the independent prognostic value of the MI Clarity M3T risk label, adjusted for clinical covariates. **C.** Kaplan–Meier analysis of overall DR comparing MI Clarity M3T risk groups in Arms B and C (intention-to-treat).


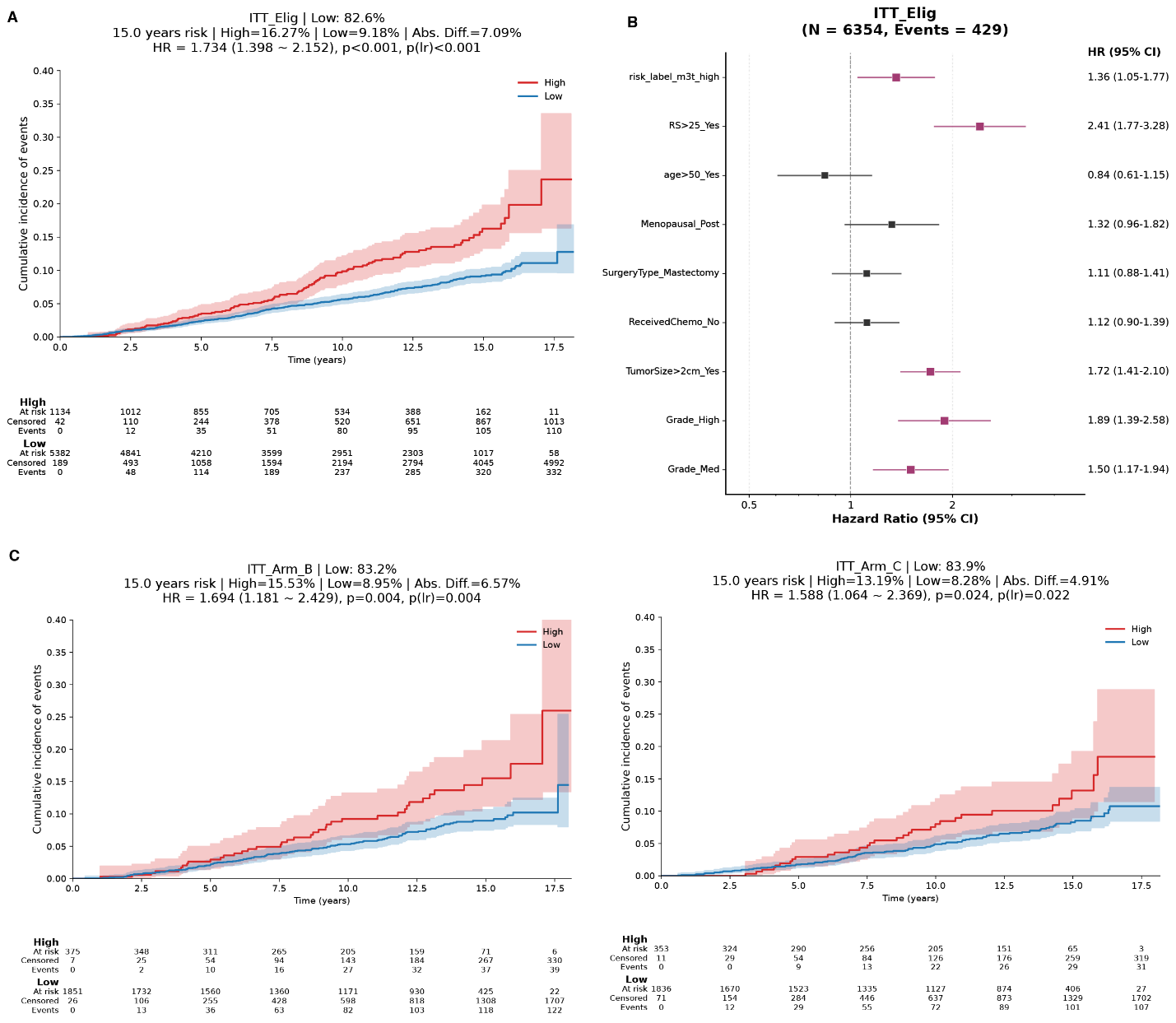

Supplement: Supplementary Figure 4 — External validation of MI Clarity M3T for overall distant recurrence (DR) in the TAILORx translational cohort. [file crc-26-0362_supplementary_figure_4_suppsf4.docx]
