# Supplementary material for: Development and Validation of a Multimodal–Multitask Deep Learning Approach for Estimating Late Distant Recurrence Risk in HR-Positive Early Breast Cancer
Source: Cancer Res Commun. 2026 Jul 31;6(7):1825–35. doi: 10.1158/2767-9764.CRC-26-0362 (PMC13425195; doi:10.1158/2767-9764.CRC-26-0362)
Supplement: Supplementary Methods [file crc-26-0362_supplementary_methods_suppsm.docx]

**Supplementary methods**

***Model development pipeline***

As outlined in the main Methods, three model variants were developed using the NSABP B-42 translational cohort.

***Image preprocessing***

WSIs from the Pramana scanner were converted from OME-TIFF format to TIFF format. Tissue masks were generated using a QuPath-based [1] pixel classifier, and HistoQC [2] was used to detect and remove coverslip edges and pen markings from the masks. Morphological transformations were applied to fill small holes and remove small objects, enhancing tissue mask quality. Tiles measuring 224×224 pixels were extracted at 10x magnification with a pixel size of ~1 micron (**Supplementary Figure 1A**).

***Feature extraction***

A pretrained CTransPath model [3] was used to generate 768-dimensional (768-D) embeddings from each tile. CTransPath uses semantically related contrastive learning to improve feature representation by leveraging diverse positive samples. The backbone architecture includes a convolutional neural network (CNN) module and a Swin transformer module. The CNN stabilizes training, while the Swin transformer extracts features through local window attention and shift-window-based self-attention mechanisms.

***Transformer-based risk score prediction***

Experimental Design

A 5-fold cross-validation strategy was performed for model development (**Supplementary Figure 1B**). In each iteration, three folds were allocated for training, one for validation, and one for test. This strategy enabled the evaluation of the entire cohort as opposed to having a fixed small set for testing. Given the small number of DR events (n = 122) in the B-42 translational cohort, stratified sampling was applied to balance DR events across the five folds. Within each test fold, patients were categorized into high- and low-risk groups using the median risk score from the corresponding training cases as the cutoff.

Model Architecture

The encoder-decoder transformer architecture [4] was used to predict WSI-level risk scores from tile-level embeddings generated by CTransPath. For the image-only model, the embeddings served as input to the transformer. For the multimodal model, clinical features (age at NSABP B-42 randomization, pathological node status, and surgery type) were concatenated with each tile embedding using an early fusion strategy, which outperformed late fusion methods (e.g., concatenation or TensorFusion [5]). The architecture included a projection layer to reduce embedding dimensionality from 768 to 256, enabling memory-efficient processing. Encoded embeddings were passed through the transformer decoder, which generated a 256-D regression token. A fully connected layer then predicted the WSI-level risk score from this token. For the M3T model, an auxiliary task of classifying lowest bone mineral density in the lumbosacral spine, total hip, or femoral neck (BMD T-score) was applied. This auxiliary task was selected because it provided greater subgroup stratification in terms of the absolute benefit of ELT and addressed practical concerns related to missing data when used as a direct input. For this purpose, following a similar strategy described by El et al. in 2024 [6], an additional classification token was used to decode the encoded tokens, and the corresponding decoded tokens were processed by an additional MLP layer to predict the lowest BMD T-score status (**Supplementary Figure 1C, D**).

Model Training

All three model variants were trained using the AdamW optimizer with a batch size of 64 and 50 epochs. The Cox partial likelihood loss function [7] was used for training. To reduce overfitting, early stopping, L1/L2 regularization (weight = 1×10⁻³), and adaptive learning rates were applied. The learning rate was set to 1×10⁻⁶ for the image-only model, and 2×10⁻⁶ for the multimodal and the M3T models. During training, 64 randomly selected tiles were used for each WSI within each epoch, while all tiles were utilized for validation and testing. Optimal epochs were selected based on the maximum concordance index (C-index) value on the validation split. For task balancing in the M3T model training, pcgrad [8], gradient-based balancing was used, which outperformed cagrad [9] and graddrop [10] method in terms of C-index on the validation split.

Model performance assessment

Model performance was assessed using internal five-fold cross-validation in the NSABP B-42 translational cohort and further evaluated through independent external validation in the TAILORx translational cohort. Risk group assignment used a prespecified cutoff derived from the training data within each cross-validation fold and was fixed for external validation. Prior to external validation, the final models were trained using the same training configuration from the cross-validation training and evaluation but with full training dataset. The locked models were then applied to the independent TAILORx cohort without retraining or recalibration.

***Statistical analysis***

Evaluation in NSABP B-42 translational cohort

We assessed the representativeness of the translational cohort compared to the full NSABP B-42 population using chi-square tests on baseline clinicopathologic variables.

The primary endpoint for model evaluation was distant recurrence (DR), defined as time from NSABP B-42 randomization to distant recurrence of breast cancer. Predefined secondary endpoints included recurrence-free interval (RFI; time from randomization to local, regional, or distant breast cancer recurrence); DFS (time from randomization to breast cancer recurrence, second primary cancer, or death); and BCFI (time from randomization to breast cancer recurrence or contralateral breast cancer as a first event. All time-to-event endpoints were measured from the date of randomization in the B-42 trial to the date of diagnosis of the specified event). Patients who were otherwise event free were censored at the date of last clinical follow-up. In addition, for BCFI, other second primary cancers and death without evidence of recurrence were treated as censored events.

Prognostic analysis in B-42

Differences between high- and low-risk groups were assessed via log-rank tests. HRs and 95% CIs were estimated using univariable Cox models. Kaplan−Meier estimates and 10-year absolute risk differences were calculated (ten years after trial randomization in B-42, corresponding to approximately fifteen years from initial diagnosis, given that randomization occurred after completion of about five years of adjuvant ET). For DR, a multivariable Cox model included model-predicted risk label, treatment assignment, and clinical covariates (age, surgery type, and original B-42 stratification factors: pathological node status, use of tamoxifen as a component of initial adjuvant therapy, and lowest bone mineral density in the lumbosacral spine, total hip, or femoral neck (BMD T-score). Significant interaction terms were also included. The main effect terms and interaction terms with significant effect were included in the final multivariable Cox model. Violation of proportional hazard (PH) assumption was checked for the main-effect-only model and clinical variables violating PH assumption were used as stratification factors in the full model with interaction. Treatment and risk label satisfied PH assumption for all models.

Predictive analysis in B-42

To assess treatment effect (ELT vs. placebo), stratified log-rank and stratified Cox models were applied, controlling for parent trial stratification variables. Analyses were performed for the full translational cohort and within high-risk and low-risk groups. Likelihood ratio tests evaluated interactions between treatment and clinical variables or risk labels. If the clinical covariates used in the interaction overlapped with stratification factors, it was removed from stratification.

In cases where the PH assumption was violated by the treatment variable, we implemented two approaches: 1) A change point (~4 years post-randomization) was defined based on relative risk in the entire cohort.[11] Separate Cox models were performed for 0–4 and >4 years. 2) We fit time-dependent Cox models including a time-by-treatment interaction term. This enabled estimation of time-varying treatment effects and treatment-by-risk interaction. Kaplan−Meier curves illustrated treatment benefit, with absolute benefit defined as the difference in 10-year Kaplan−Meier estimate of event risk between ELT and placebo treatments.

Model Calibration robustness assessment

Calibration of predicted risk was evaluated at prespecified time horizons. Models were trained using Cox partial likelihood and produced continuous risk scores representing relative hazard. Absolute risk estimates were derived by fitting a Cox proportional hazards model with the model-derived risk score as the sole covariate to estimate the baseline survival function, from which predicted 10-year survival probabilities and cumulative risks were obtained. Observed event probabilities were estimated using the Kaplan–Meier method within strata defined by quantiles of predicted risk. Calibration was summarized using calibration slope, calibration-in-the-large, expected calibration error, and maximum bin-level error.

To assess robustness, calibration analyses were repeated over 30 random splits of the dataset. For each split, 50% of samples were used to estimate the baseline survival function and the remaining 50% were used for calibration evaluation. Five risk strata were defined based on quantiles of predicted risk, ensuring a minimum of 10 events per stratum for stable estimation. Calibration metrics were summarized across repeats.

External validation in TAILORx translational cohort

Overall distant recurrence (DR) prognostication was evaluated using distant recurrence–free interval (DRFI), defined as the time from TAILORx registration to distant recurrence of breast cancer, or death attributed to distant recurrence if death was the first manifestation [12]. In B-42 DR endpoint was defined from the time of randomization to development of distant recurrence. However, in B-42 randomization occurred after patients had completed about 5 years of endocrine therapy and were disease-free.

For overall DR analyses, cumulative incidence of DR events was estimated using the Kaplan–Meier method, and log-rank tests and univariable Cox models compared overall DR between MI Clarity M3T risk groups. A multivariable Cox model evaluated the independent contribution of MI Clarity M3T risk labels, adjusting for age (>50 vs ≤50 years), menopausal status (postmenopausal vs premenopausal), surgery (mastectomy vs lumpectomy), chemotherapy receipt (no vs yes), tumor size (>2 cm vs ≤2 cm), tumor grade (high/med vs low), and Oncotype DX RS (>25 vs 0–25).

For late DR analyses, to align with the B-42 design, analyses were restricted to the subset of 4,300 TAILORx patients who had completed ≥4.5 years of ET, were disease-free at the 5-year landmark. The 4.5-year threshold was chosen to account for minor variations in treatment timing and to ensure adequate sample size for analysis. Among 4,300 patients, the median duration of ET was 6.0 years (interquartile range, 5.1–8.5; maximum, 18.1 years), and 85.7% received more than 5 years of ET. Kaplan–Meier analyses and Cox models were performed in the late distant recurrence cohort.

***Model interpretation analysis***

To elucidate image-derived predictive patterns, WSI-level heatmaps were generated using the trained image-only model to visualize the spatial distribution of model attention, patch-level risk, and attention-weighted contribution scores. Attention heatmaps were generated using self-attention values from the second decoder block of the image-only model. These attention scores identified tissue regions prioritized by the model during image-based WSI-level prediction.

Patch-level risk score heatmaps were generated by passing each tile individually through the trained image-only network and mapping the resulting single-tile risk score back to the tile’s original spatial coordinate on the WSI. Contribution heatmaps were derived by multiplying the single-tile risk score by the corresponding attention score for each tile. Thus, contribution scores integrated both the local recurrence-associated risk signal and the model’s attention to that region, highlighting tiles with the greatest influence on the final image-based WSI-level prediction. The contribution scores were strongly correlated with the image-only model’s predicted WSI-level risk scores when all tiles were used, supporting their use for spatial interpretation.

To further explore morphologic features associated with image-derived risk, we analyzed 20 representative WSIs, including the 10 slides with the highest image-only predicted risk scores and the 10 slides with the lowest image-only predicted risk scores. Tile-level CTransPath features were extracted as 768-dimensional embeddings. These tile-level embeddings were clustered using K-means and visualized using UMAP. The resulting clusters were reviewed to identify morphologically coherent groups of tiles. Patch-level risk scores were then compared across clusters to determine whether specific morphologic patterns were associated with higher recurrence-related signal.

For representative multimodal use-case analyses, heatmaps were generated separately using the image-only model and the M3T model. These examples were used to illustrate how integration of clinicopathologic features with image-derived representations altered model risk classification and spatial risk-contribution patterns. In the clinically low-risk use case, M3T-derived heatmaps were used to assess whether high-risk and high-contribution regions localized to tumor-bearing tissue despite favorable clinicopathologic features.

**References**

1. Bankhead, P., et al., *QuPath: Open source software for digital pathology image analysis.* Sci Rep, 2017. **7**(1): p. 16878.

2. Janowczyk, A., et al., *HistoQC: An Open-Source Quality Control Tool for Digital Pathology Slides.* JCO Clin Cancer Inform, 2019. **3**: p. 1-7.

3. Wang, X., et al., *Transformer-based unsupervised contrastive learning for histopathological image classification.* Med Image Anal, 2022. **81**: p. 102559.

4. Vaswani, A., et al. *Attention Is All You Need*. 2017. arXiv:1706.03762 DOI: 10.48550/arXiv.1706.03762.

5. Zadeh, A., et al. *Tensor Fusion Network for Multimodal Sentiment Analysis*. 2017. arXiv:1707.07250 DOI: 10.48550/arXiv.1707.07250.

6. El Nahhas, O.S.M., et al. *Joint Multi-task Learning Improves Weakly-Supervised Biomarker Prediction in Computational Pathology*. in *Medical Image Computing and Computer Assisted Intervention – MICCAI 2024*. 2024. Cham: Springer Nature Switzerland.

7. Katzman, J.L., et al., *DeepSurv: personalized treatment recommender system using a Cox proportional hazards deep neural network.* BMC Med Res Methodol, 2018. **18**(1): p. 24.

8. Yu, T., et al. *Gradient Surgery for Multi-Task Learning*. 2020. arXiv:2001.06782 DOI: 10.48550/arXiv.2001.06782.

9. Liu, B., et al. *Conflict-Averse Gradient Descent for Multi-task Learning*. 2021. arXiv:2110.14048 DOI: 10.48550/arXiv.2110.14048.

10. Chen, Z., et al. *Just Pick a Sign: Optimizing Deep Multitask Models with Gradient Sign Dropout*. 2020. arXiv:2010.06808 DOI: 10.48550/arXiv.2010.06808.

11. Klein, J. and M. Moeschberger, *Survival analysis: techniques for censored and truncated data*, in *Statistics for Biology and Health*. 2003.

12. Sparano, J.A., et al., *Adjuvant Chemotherapy Guided by a 21-Gene Expression Assay in Breast Cancer.* N Engl J Med, 2018. **379**(2): p. 111-121.
