# Supplementary material for: Development and Validation of a Multimodal–Multitask Deep Learning Approach for Estimating Late Distant Recurrence Risk in HR-Positive Early Breast Cancer
Source: Cancer Res Commun. 2026 Jul 31;6(7):1825–35. doi: 10.1158/2767-9764.CRC-26-0362 (PMC13425195; doi:10.1158/2767-9764.CRC-26-0362)
Supplement: Supplementary Table 1 — Clinicopathologic and treatment characteristics of patients in the translational cohort versus remaining NSABP B-42 patients. [file crc-26-0362_supplementary_table_1_suppst1.docx]

**Supplementary Table 1. Clinicopathologic and treatment characteristics of patients in the translational cohort versus remaining NSABP B-42 patients.**

| **Characteristics** | | **Translational B-42 No. (%)** | **Overall B-42 No. (%)** | **Remaining B42-No. (%)** | **Chi-square *P* value (Translational vs Remaining) ^a^** |
| --- | --- | --- | --- | --- | --- |
| **Age at randomization** | | | | | 0.05 |
|  | **<60 yrs.** | 753 (33.2) | 1344 (34.4) | 591 (36.2) |  |
|  | **≥60 yrs.** | 1518 (66.8) | 2559 (65.6) | 1041 (63.8) |  |
| **Pathological node status** | | | | | <0.01 |
|  | **Negative** | 1365 (60.1) | 2240 (57.4) | 875 (53.6) |  |
|  | **Positive** | 906 (39.9) | 1663 (42.6) | 757 (46.4) |  |
| **Lowest BMD T-score** | | | | | 0.98 |
|  | **≤-2.0** | 556 (24.5) | 954 (24.4) | 398 (24.4) |  |
|  | **>-2.0** | 1715 (75.5) | 2949 (75.6) | 1234 (75.6) |  |
| **Prior tamoxifen** | | | | | 0.27 |
|  | **No** | 1400 (61.6) | 2377 (60.9) | 977 (59.9) |  |
|  | **Yes** | 871 (38.4) | 1526 (39.1) | 655 (40.1) |  |
| **HER2 status** | | | | | 0.03 |
|  | **Negative** | 1802 (79.3) | 3041 (77.9) | 1239 (75.9) |  |
|  | **Positive** | 310 (13.7) | 559 (14.3) | 249 (15.3) |  |
|  | **Unknown** | 159 (7.0) | 303 (7.8) | 144 (8.8) |  |
| **Surgery type** | | | | | 0.25 |
|  | **Lumpectomy** | 1399 (61.6) | 2374 (60.8) | 975 (59.7) |  |
|  | **Mastectomy** | 872 (38.4) | 1529 (39.2) | 657 (40.3) |  |
| **Treatment** | | | | | 0.79 |
|  | **Placebo** | 1141 (50.2) | 1953 (50.0) | 812 (49.8) |  |
|  | **Letrozole** | 1130 (49.8) | 1950 (50.0) | 820 (50.2) |  |

^a^ *P* values comparing the translational cohort versus the remaining B-42 patients were calculated using the Chi-square test.
