# Supplementary material for: Development and Validation of a Multimodal–Multitask Deep Learning Approach for Estimating Late Distant Recurrence Risk in HR-Positive Early Breast Cancer
Source: Cancer Res Commun. 2026 Jul 31;6(7):1825–35. doi: 10.1158/2767-9764.CRC-26-0362 (PMC13425195; doi:10.1158/2767-9764.CRC-26-0362)
Supplement: Supplementary Table 2 — Prognostication performance comparison of models (image-only, multimodal, M3T model) for risk of distant recurrence (DR). [file crc-26-0362_supplementary_table_2_suppst2.docx]

**Supplementary Table 2. Prognostication performance comparison of models (image-only, multimodal, M3T model) for risk of distant recurrence (DR).**

| **Model** | **Group** | **Low-risk 10-yr DR estimate (%)** | **High-risk 10-yr DR estimate (%)** | **Absolute difference (%)** | **HR (95% CI)** | ***P* value** |
| --- | --- | --- | --- | --- | --- | --- |
| **Image**  **only** | **All Patients** | 2.72 | 8.5 | 5.79 | 3.419 (2.241–5.215) | <0.001 |
|  | **ELT** | 2.28 | 6.81 | 4.54 | 3.206 (1.664–6.174) | <0.001 |
|  | **Placebo** | 3.18 | 10.11 | 6.94 | 3.571 (2.053–6.211) | <0.001 |
| **Multi modal** | **All Patients** | 2.09 | 9.22 | 7.13 | 4.507 (2.863–7.096) | <0.001 |
|  | **ELT** | 1.65 | 7.38 | 5.73 | 4.577 (2.212–9.470) | <0.001 |
|  | **Placebo** | 2.52 | 11.06 | 8.54 | 4.529 (2.531–8.104) | <0.001 |
| **M3T** | **All Patients** | 1.69 | 9.63 | 7.95 | 5.710 (3.500–9.317) | <0.001 |
|  | **ELT** | 1.44 | 7.59 | 6.15 | 5.300 (2.475–11.349) | <0.001 |
|  | **Placebo** | 1.93 | 11.68 | 9.75 | 6.109 (3.220–11.593) | <0.001 |
