# Supplementary material for: Development and Validation of a Multimodal–Multitask Deep Learning Approach for Estimating Late Distant Recurrence Risk in HR-Positive Early Breast Cancer
Source: Cancer Res Commun. 2026 Jul 31;6(7):1825–35. doi: 10.1158/2767-9764.CRC-26-0362 (PMC13425195; doi:10.1158/2767-9764.CRC-26-0362)
Supplement: Supplementary Table 3 — Results of the multivariable analysis of DR for the image-only model. [file crc-26-0362_supplementary_table_3_suppst3.docx]

**Supplementary Table 3. Results of the multivariable analysis of DR for the image-only model.**

| **Covariate** | **Level** | **HR [95% CI]** | ***P* value** |
| --- | --- | --- | --- |
| **Treatment** | Placebo | 1 | 0.017 |
|  | ELT | 0.642 (0.446–0.923) |  |
| **Image-only risk group** | Low | 1 | <0.001 |
|  | High | 2.761 (1.799–4.238) |  |
| **Pathological node status** | Negative | 1 | <0.001 |
|  | Positive | 2.324 (1.573-3.435) |  |
| **Surgery type** | Lumpectomy | 1 | <0.001 |
|  | Mastectomy | 2.146 (1.479-3.115) |  |
