# Supplementary material for: Development and Validation of a Multimodal–Multitask Deep Learning Approach for Estimating Late Distant Recurrence Risk in HR-Positive Early Breast Cancer
Source: Cancer Res Commun. 2026 Jul 31;6(7):1825–35. doi: 10.1158/2767-9764.CRC-26-0362 (PMC13425195; doi:10.1158/2767-9764.CRC-26-0362)
Supplement: Supplementary Table 4 — Results of the multivariable analysis of DR for the M3T model. [file crc-26-0362_supplementary_table_4_suppst4.docx]

**Supplementary Table 4. Results of the multivariable analysis of DR for the M3T model.**

| **Covariate** | **Level** | **HR [95% CI]** | ***P* value** |
| --- | --- | --- | --- |
| **Treatment** | Placebo | 1 | 0.011 |
|  | ELT | 0.620 (0.431–0.891) |  |
| **M3T model risk group** | Low | 1 | <0.001 |
|  | High | 3.575 (2.039–6.269) |  |
| **Pathological node status** | Negative | 1 | 0.008 |
|  | Positive | 1.707 (1.126–2.586) |  |
| **Surgery type** | Lumpectomy | 1 | 0.011 |
|  | Mastectomy | 1.608 (1.096–2.360) |  |
