# Supplementary material for: Development and Validation of a Multimodal–Multitask Deep Learning Approach for Estimating Late Distant Recurrence Risk in HR-Positive Early Breast Cancer
Source: Cancer Res Commun. 2026 Jul 31;6(7):1825–35. doi: 10.1158/2767-9764.CRC-26-0362 (PMC13425195; doi:10.1158/2767-9764.CRC-26-0362)
Supplement: Supplementary Table 5 — Prognostic performance of MI Clarity M3T model for breast cancer-free interval (BCFI), disease-free survival (DFS) and recurrence-free interval (RFI). [file crc-26-0362_supplementary_table_5_suppst5.docx]

**Supplementary Table 5. Prognostic performance of MI Clarity M3T model for breast cancer-free interval (BCFI), disease-free survival (DFS) and recurrence-free interval (RFI).**

| **Clinical endpoints** | **Group** | **Low-risk 10-yr event estimate (%)** | **High-risk 10-yr event estimate (%)** | **Absolute difference (%)** | **HR (95% CI)** | ***P* value** |
| --- | --- | --- | --- | --- | --- | --- |
| **BCFI** | **All Patients** | 8.68 | 15.74 | 7.06 | 1.921 [1.479–2.496] | <0.001 |
|  | **ELT** | 7.34 | 13.52 | 6.19 | 2.015 [1.340–3.030] | <0.001 |
|  | **Placebo** | 9.99 | 17.97 | 7.98 | 1.881 [1.335–2.650] | <0.001 |
| **DFS** | **All Patients** | 22.16 | 28.93 | 6.77 | 1.398 [1.180–1.655] | <0.001 |
|  | **ELT** | 19.7 | 26.37 | 6.67 | 1.417 [1.100–1.825] | 0.007 |
|  | **Placebo** | 24.6 | 31.49 | 6.89 | 1.391 [1.108–1.745] | 0.004 |
| **RFI** | **All Patients** | 3.52 | 12.26 | 8.75 | 3.611 [2.514–5.188] | <0.001 |
|  | **ELT** | 3.32 | 10.74 | 7.42 | 3.422 [1.988–5.888] | <0.001 |
|  | **Placebo** | 3.71 | 13.78 | 10.07 | 3.895 [2.375–6.389] | <0.001 |
