# Supplementary material for: Development and Validation of a Multimodal–Multitask Deep Learning Approach for Estimating Late Distant Recurrence Risk in HR-Positive Early Breast Cancer
Source: Cancer Res Commun. 2026 Jul 31;6(7):1825–35. doi: 10.1158/2767-9764.CRC-26-0362 (PMC13425195; doi:10.1158/2767-9764.CRC-26-0362)
Supplement: Supplementary Table 6 — Clinicopathologic, and treatment characteristics of patients classified as MI Clarity–High versus Low risk in the NSABP B-42 translational cohort. [file crc-26-0362_supplementary_table_6_suppst6.docx]

**Supplementary Table 6. Clinicopathologic, and treatment characteristics of patients classified as MI Clarity–High versus Low risk in the NSABP B-42 translational cohort.**

| **Variable** | **Category** | **MI Clarity-High No. (%)** | **MI Clarity -Low No. (%)** | **Total** | ***P* value (MI Clarity High vs Low)** |
| --- | --- | --- | --- | --- | --- |
| **Total** |  | 1133 (49.9%) | 1138 (50.1%) | 2271 |  |
| **AGE** | <60 | 402 (35.5%) | 351 (30.8%) | 753 (33.2%) | 0.021 |
|  | ≥60 | 731 (64.5%) | 787 (69.2%) | 1518 (66.8%) |  |
| **Pathological node status** | Negative | 396 (35.0%) | 969 (85.1%) | 1365 (60.1%) | <0.001 |
|  | Positive | 737 (65.0%) | 169 (14.9%) | 906 (39.9%) |  |
| **Lowest BMD T-score** | ≤-2.0 | 300 (26.5%) | 256 (22.5%) | 556 (24.5%) | 0.031 |
|  | >-2.0 | 833 (73.5%) | 882 (77.5%) | 1715 (75.5%) |  |
| **Prior tamoxifen** | No | 690 (60.9%) | 710 (62.4%) | 1400 (61.6%) | 0.492 |
|  | Yes | 443 (39.1%) | 428 (37.6%) | 871 (38.4%) |  |
| **HER2** | Negative | 887 (78.3%) | 915 (80.4%) | 1802 (79.3%) | 0.086 |
|  | Positive | 172 (15.2%) | 138 (12.1%) | 310 (13.7%) |  |
|  | Unknown | 74 (6.5%) | 85 (7.5%) | 159 (7.0%) |  |
| **Surgery type** | Lumpectomy | 467 (41.2%) | 932 (81.9%) | 1399 (61.6%) | <0.001 |
|  | Mastectomy | 666 (58.8%) | 206 (18.1%) | 872 (38.4%) |  |
| **Treatment** | Placebo | 568 (50.1%) | 573 (50.4%) | 1141 (50.2%) | 0.95 |
|  | Letrozole | 565 (49.9%) | 565 (49.6%) | 1130 (49.8%) |  |

Note: Risk labels were generated by the M3T model. *P* values were calculated using the chi-square test for categorical variables.
