# Supplementary material for: Development and Validation of a Multimodal–Multitask Deep Learning Approach for Estimating Late Distant Recurrence Risk in HR-Positive Early Breast Cancer
Source: Cancer Res Commun. 2026 Jul 31;6(7):1825–35. doi: 10.1158/2767-9764.CRC-26-0362 (PMC13425195; doi:10.1158/2767-9764.CRC-26-0362)
Supplement: Supplementary Table 7 — Calibration performance of models for 10-year distant recurrence risk in the NSABP B-42 translational cohort. [file crc-26-0362_supplementary_table_7_suppst7.docx]

**Supplementary Table 7. Calibration performance of models for 10-year distant recurrence risk in the NSABP B-42 translational cohort.**

|  | **Image-only Model** | | **Multimodal Model** | | **M3T Model** | |
| --- | --- | --- | --- | --- | --- | --- |
| **Metrics** | **Mean + SD** | **Median [2.5%, 97.5%]** | **Mean + SD** | **Median [2.5%, 97.5%]** | **Mean + SD** | **Median [2.5%, 97.5%]** |
| **Observed risk** | 0.055 ± 0.004 | 0.055 [0.047, 0.062] | 0.055 ± 0.004 | 0.055 [0.047, 0.062] | 0.055 ± 0.004 | 0.055 [0.047, 0.062] |
| **Mean predicted risk** | 0.057 ± 0.005 | 0.058 [0.049, 0.068] | 0.058 ± 0.005 | 0.058 [0.049, 0.066] | 0.057 ± 0.005 | 0.057 [0.049, 0.067] |
| **Calibration-in-the-large** | 0.002 ± 0.009 | 0.003 [-0.013, 0.019] | 0.002 ± 0.008 | 0.001 [-0.013, 0.017] | 0.002 ± 0.008 | 0.001 [-0.013, 0.017] |
| **ECE** | 0.015 ± 0.005 | 0.015 [0.006, 0.028] | 0.012 ± 0.005 | 0.012 [0.005, 0.025] | 0.011 ± 0.005 | 0.010 [0.004, 0.024] |
| **Max bin error** | 0.032 ± 0.015 | 0.029 [0.015, 0.068] | 0.029 ± 0.019 | 0.024 [0.010, 0.085] | 0.029 ± 0.019 | 0.021 [0.009, 0.080] |
| **Calibration slope** | 1.013 ± 0.216 | 0.999 [0.616, 1.387] | 1.042 ± 0.181 | 1.020 [0.766, 1.347] | 1.066 ± 0.207 | 1.072 [0.675, 1.436] |
