# Supplementary material for: Development and Validation of a Multimodal–Multitask Deep Learning Approach for Estimating Late Distant Recurrence Risk in HR-Positive Early Breast Cancer
Source: Cancer Res Commun. 2026 Jul 31;6(7):1825–35. doi: 10.1158/2767-9764.CRC-26-0362 (PMC13425195; doi:10.1158/2767-9764.CRC-26-0362)
Supplement: Supplementary Table 8 — Performance comparison of models (image-only, multimodal, M3T model) for predicting benefit from ELT. [file crc-26-0362_supplementary_table_8_suppst8.docx]

**Supplementary Table 8. Performance comparison of models (image-only, multimodal, M3T model) for predicting benefit from ELT.**

| **Model** | **Risk Group** | **ELT 10-yr DR estimate (%)** | **Placebo 10-yr DR estimate (%)** | **Absolute benefit (%)** | **HR (95% CI)** | ***P* value^a^** | ***P* interaction^b^** |
| --- | --- | --- | --- | --- | --- | --- | --- |
|  | **All Patients** | 4.52 | 6.75 | 2.23 | 0.621 (0.432–0.894) | 0.01 |  |
| **Image-**  **only** | **Low** | 2.28 | 3.18 | 0.9 | 0.581 (0.271–1.247) | 0.159 | 0.868 |
|  | **High** | 6.81 | 10.11 | 3.3 | 0.670 (0.442–1.017) | 0.058 |  |
| **Multi modal** | **Low** | 1.65 | 2.52 | 0.87 | 0.575 (0.247–1.342) | 0.196 | 0.857 |
|  | **High** | 7.38 | 11.06 | 3.68 | 0.644 (0.430–0.965) | 0.032 |  |
| **M3T** | **Low** | 1.44 | 1.93 | 0.49 | 0.664 (0.266–1.659) | 0.378 | 0.817 |
|  | **High** | 7.59 | 11.68 | 4.09 | 0.614 (0.413–0.913) | 0.015 |  |

^a^ *P* value from the log-rank test.

^b^ Interaction *P* value for the treatment x risk label term in each model.
