# Supplementary material for: Development and Validation of a Multimodal–Multitask Deep Learning Approach for Estimating Late Distant Recurrence Risk in HR-Positive Early Breast Cancer
Source: Cancer Res Commun. 2026 Jul 31;6(7):1825–35. doi: 10.1158/2767-9764.CRC-26-0362 (PMC13425195; doi:10.1158/2767-9764.CRC-26-0362)
Supplement: Supplementary Table 9 — Predictive performance of the MI Clarity M3T model for ELT benefit across clinical subgroups. [file crc-26-0362_supplementary_table_9_suppst9.docx]

**Supplementary Table 9. Predictive performance of the MI Clarity M3T model for ELT benefit across clinical subgroups.**

| **Subgroup** | | **Risk Group** | **ELT 10-yr DR estimate (%)** | **Placebo 10-yr DR estimate (%)** | **Absolute benefit (%)** | **HR (95% CI)** | ***P* value** | ***P* interaction** |
| --- | --- | --- | --- | --- | --- | --- | --- | --- |
| **Age at**  **randomization** | **≤60** | **All Patients** | 3.17 | 7.66 | 4.49 | 0.423 (0.220–0.813) | 0.008 | 0.719 |
|  |  | **Low** | 0.64 | 2.1 | 1.46 | 0.222 (0.025–2.016) | 0.144 |  |
|  |  | **High** | 5.26 | 12.71 | 7.46 | 0.434 (0.218–0.861) | 0.014 |  |
|  | **>60** | **All Patients** | 5.38 | 6.23 | 0.85 | 0.772 (0.493–1.209) | 0.257 | 0.977 |
|  |  | **Low** | 1.88 | 1.85 | -0.03 | 0.840 (0.282–2.505) | 0.754 |  |
|  |  | **High** | 9.29 | 11.01 | 1.72 | 0.809 (0.494–1.325) | 0.399 |  |
| **HER2 status** | **Neg.** | **All Patients** | 4.89 | 7.02 | 2.13 | 0.635 (0.428–0.941) | 0.023 | 0.731 |
|  |  | **Low** | 1.25 | 1.95 | 0.7 | 0.519 (0.174–1.554) | 0.233 |  |
|  |  | **High** | 8.57 | 12.36 | 3.8 | 0.653 (0.428–0.997) | 0.047 |  |
|  | **Pos.** | **All Patients** | 3.64 | 6.85 | 3.22 | 0.420 (0.130–1.354) | 0.135 | 0.212^a^ |
|  |  | **Low** | 3.73 | 1.47 | -2.26 | 1.961 (0.177–21.738) | 0.576 |  |
|  |  | **High** | 3.48 | 10.97 | 7.49 | 0.267 (0.057–1.244) | 0.071 |  |
| **Prior tamoxifen** | **No** | **All Patients** | 4.82 | 7.09 | 2.27 | 0.642 (0.404–1.019) | 0.058 | 0.410^a^ |
|  |  | **Low** | 1.34 | 2.63 | 1.29 | 0.395 (0.121–1.287) | 0.111 |  |
|  |  | **High** | 8.49 | 11.5 | 3.01 | 0.716 (0.432–1.186) | 0.193 |  |
|  | **Yes** | **All Patients** | 4.1 | 6.26 | 2.16 | 0.589 (0.327–1.063) | 0.075 | 0.113^a^ |
|  |  | **Low** | 1.61 | 0.91 | -0.7 | 1.962 (0.355–10.856) | 0.432 |  |
|  |  | **High** | 6.34 | 12 | 5.66 | 0.483 (0.253–0.922) | 0.024 |  |
| **Pathological node status** | **Neg.** | **All Patients** | 3.01 | 2.95 | -0.06 | 0.915 (0.491–1.704) | 0.78 | 0.634 |
|  |  | **Low** | 1.47 | 1.8 | 0.32 | 0.742 (0.275–2.002) | 0.555 |  |
|  |  | **High** | 6.74 | 5.88 | -0.87 | 1.030 (0.462–2.297) | 0.941 |  |
|  | **Pos.** | **All Patients** | 6.73 | 12.67 | 5.94 | 0.511 (0.325–0.803) | 0.003 | 0.892^a^ |
|  |  | **Low** | 1.23 | 2.73 | 1.5 | 0.362 (0.033–4.025) | 0.389 |  |
|  |  | **High** | 8 | 14.83 | 6.83 | 0.521 (0.328–0.826) | 0.005 |  |
| **Surgery type** | **Lump-ectomy** | **All Patients** | 3.3 | 3.74 | 0.44 | 0.827 (0.465–1.471) | 0.518 | 0.112 |
|  |  | **Low** | 1.19 | 2.38 | 1.19 | 0.415 (0.144–1.199) | 0.094 |  |
|  |  | **High** | 7.79 | 6.24 | -1.55 | 1.269 (0.621–2.594) | 0.513 |  |
|  | **Mast-ectomy** | **All Patients** | 6.4 | 11.9 | 5.5 | 0.506 (0.315–0.813) | 0.004 | 0.007 |
|  |  | **Low** | 2.67 | 0 | -2.67 | 25022372.142 (0.000–Inf) | 0.089 |  |
|  |  | **High** | 7.4 | 16.09 | 8.69 | 0.437 (0.267–0.715) | <0.001 |  |
| **Lowest BMD T-score** | **≤ -2.0** | **All Patients** | 4.19 | 9.95 | 5.76 | 0.367 (0.182–0.741) | 0.004 | 0.055^a^ |
|  |  | **Low** | 0 | 3.63 | 3.63 | 0.000 (0.000–Inf) | 0.013 |  |
|  |  | **High** | 7.61 | 15.58 | 7.97 | 0.469 (0.227–0.967) | 0.036 |  |
|  | **>-2.0** | **All Patients** | 4.63 | 5.74 | 1.11 | 0.775 (0.503–1.193) | 0.245 | 0.301 |
|  |  | **Low** | 1.84 | 1.43 | -0.4 | 1.270 (0.439–3.675) | 0.659 |  |
|  |  | **High** | 7.6 | 10.34 | 2.74 | 0.694 (0.431–1.117) | 0.13 |  |

^a^ log(time) transformation was applied to model the time-varying effect.
