# Supplementary material for: Development and Validation of a Multimodal–Multitask Deep Learning Approach for Estimating Late Distant Recurrence Risk in HR-Positive Early Breast Cancer
Source: Cancer Res Commun. 2026 Jul 31;6(7):1825–35. doi: 10.1158/2767-9764.CRC-26-0362 (PMC13425195; doi:10.1158/2767-9764.CRC-26-0362)
Supplement: Supplementary Table 10 — Time-dependent predictive performance of the M3T model for ELT benefit in clinical subgroups with violated proportional hazards (PH) assumption. [file crc-26-0362_supplementary_table_10_suppst10.docx]

**Supplementary Table 10. Time-dependent predictive performance of the M3T model for ELT benefit in clinical subgroups with violated proportional hazards (PH) assumption.**

| **Subgroup** | | **Risk group** | **Time period** | **Low-risk 10-yr DR estimate (%)** | **High-risk 10-yr DR estimate (%)** | **Absolute difference (%)** | **HR (95% CI)** | ***P* value** |
| --- | --- | --- | --- | --- | --- | --- | --- | --- |
| **HER2 status** | **Pos.** | **All Pts.** | ≤4 yrs. | 0.69 | 3.91 | 3.22 | 0.171 (0.020–1.439) | 0.104 |
|  |  | **All Pts.** | >4 yrs. | 2.52 | 2.73 | 0.21 | 0.807 (0.178–3.659) | 0.781 |
|  |  | **Low^a^** | ≤4 yrs. | 1.54 | 1.47 | -0.07 | 0.000 (0.000–0.000) | <0.001 |
|  |  | **Low** | >4 yrs. | 1.82 | 0 | -1.82 | 0.000 (0.000–0.000) | <0.001 |
|  |  | **High** | ≤4 yrs. | 0 | 5.82 | 5.82 | 0.000 (0.000–Inf) | 0.999 |
|  |  | **High** | >4 yrs. | 3.06 | 4.75 | 1.7 | 0.616 (0.111–3.424) | 0.579 |
| **Prior Tamoxifen** | **No** | **All Pts.** | ≤4 yrs. | 2.4 | 2.79 | 0.39 | 0.830 (0.427–1.615) | 0.584 |
|  |  | **All Pts.** | >4 yrs. | 2.18 | 3.88 | 1.7 | 0.508 (0.265–0.973) | 0.041 |
|  |  | **Low^a^** | ≤4 yrs. | 0.9 | 1.18 | 0.28 | 0.679 (0.151–3.043) | 0.613 |
|  |  | **Low** | >4 yrs. | 0.38 | 1.31 | 0.92 | 0.174 (0.020–1.497) | 0.111 |
|  |  | **High** | ≤4 yrs. | 3.99 | 4.38 | 0.39 | 0.883 (0.420–1.856) | 0.742 |
|  |  | **High** | >4 yrs. | 4.1 | 6.41 | 2.31 | 0.600 (0.300–1.199) | 0.148 |
| **Prior Tamoxifen** | **Yes** | **All Pts.^a^** | ≤4 yrs. | 2.55 | 2.83 | 0.28 | 0.916 (0.404–2.077) | 0.834 |
|  |  | **All Pts.** | >4 yrs. | 1.44 | 3.19 | 1.75 | 0.371 (0.153–0.898) | 0.028 |
|  |  | **Low** | ≤4 yrs. | 0.5 | 0.91 | 0.42 | 0.546 (0.050–6.027) | 0.622 |
|  |  | **Low** | >4 yrs. | 1.05 | 0 | -1.05 | 205299422.803 (0.000–Inf) | 0.998 |
|  |  | **High^a^** | ≤4 yrs. | 4.4 | 4.83 | 0.43 | 0.970 (0.404–2.332) | 0.947 |
|  |  | **High** | >4 yrs. | 1.79 | 6.57 | 4.78 | 0.211 (0.071–0.627) | 0.005 |
| **Pathological node status** | **Pos.** | **All Pts.** | ≤4 yrs. | 3.84 | 5.29 | 1.45 | 0.720 (0.385–1.348) | 0.305 |
|  |  | **All Pts.** | >4 yrs. | 2.67 | 6.6 | 3.94 | 0.358 (0.183–0.700) | 0.003 |
|  |  | **Low^a^** | ≤4 yrs. | 1.23 | 1.28 | 0.05 | 0.000 (0.000–0.000) | <0.001 |
|  |  | **Low** | >4 yrs. | 0 | 1.3 | 1.3 | 0.000 (0.000–0.000) | <0.001 |
|  |  | **High** | ≤4 yrs. | 4.45 | 6.15 | 1.71 | 0.726 (0.381–1.382) | 0.33 |
|  |  | **High** | >4 yrs. | 3.29 | 7.78 | 4.48 | 0.374 (0.191–0.734) | 0.004 |
| **Lowest BMD T-score** | **≤-2.0** | **All Pts.^a^** | ≤4 yrs. | 1.83 | 4.08 | 2.25 | 0.428 (0.149–1.231) | 0.115 |
|  |  | **All Pts.** | >4 yrs. | 2.11 | 5.28 | 3.17 | 0.328 (0.128–0.840) | 0.02 |
|  |  | **Low** | ≤4 yrs. | 0 | 1.56 | 1.56 | 0.000 (0.000–Inf) | 0.999 |
|  |  | **Low** | >4 yrs. | 0 | 1.87 | 1.87 | 0.000 (0.000–Inf) | 0.999 |
|  |  | **High^a^** | ≤4 yrs. | 3.36 | 6.27 | 2.91 | 0.556 (0.186–1.661) | 0.293 |
|  |  | **High** | >4 yrs. | 3.91 | 8.31 | 4.4 | 0.412 (0.156–1.086) | 0.073 |

^a^ Specific clinical subgroups that violate the PH assumption.
