# Supplementary material for: Development and Validation of a Multimodal–Multitask Deep Learning Approach for Estimating Late Distant Recurrence Risk in HR-Positive Early Breast Cancer
Source: Cancer Res Commun. 2026 Jul 31;6(7):1825–35. doi: 10.1158/2767-9764.CRC-26-0362 (PMC13425195; doi:10.1158/2767-9764.CRC-26-0362)
Supplement: Supplementary Table 11 — Clinicopathologic, and treatment characteristics of patients classified as MI Clarity–High versus Low risk in the TAILORx translational late-DR cohort. [file crc-26-0362_supplementary_table_11_suppst11.docx]

**Supplementary Table 11. Clinicopathologic, and treatment characteristics of patients classified as MI Clarity–High versus Low risk in the TAILORx translational late-DR cohort.**

| **Variable** | **Category** | **MI Clarity-High No. (%)** | **MI Clarity-Low No. (%)** | **Total** | ***P* value (MI Clarity High vs Low)** |
| --- | --- | --- | --- | --- | --- |
| **Total Patients** |  | 740 (17.2%) | 3560 (82.8%) | 4300 |  |
| **Assigned treatment arm** | A (RS 0-10, assigned to endocrine therapy alone) | 142 (19.2%) | 612 (17.2%) | 754 (17.5%) | 0.255 |
|  | B (RS 11-25, assigned to endocrine therapy alone) | 270 (36.5%) | 1341 (37.7%) | 1611 (37.5%) |  |
|  | C (RS 11-25, assigned to chemoendocrine therapy alone) | 256 (34.6%) | 1313 (36.9%) | 1569 (36.5%) |  |
|  | D (RS ≥26, assigned to chemoendocrine therapy ) | 72 (9.7%) | 294 (8.3%) | 366 (8.5%) |  |
| **Age-Median (IQR)** |  | 59.0 (52.0-64.0) | 55.0 (49.0-62.0) | 56.0 (49.0-63.0) | <0.001 |
| **Menopausal status** | Post | 562 (75.9%) | 2274 (63.9%) | 2836 (66.0%) | <0.001 |
|  | Pre | 178 (24.1%) | 1286 (36.1%) | 1464 (34.0%) |  |
| **Surgery type** | Lumpectomy | 197 (26.6%) | 2896 (81.3%) | 3093 (71.9%) | <0.001 |
|  | Mastectomy | 543 (73.4%) | 664 (18.7%) | 1207 (28.1%) |  |
| **Grade** | High | 123 (16.6%) | 512 (14.4%) | 635 (14.8%) | <0.001 |
|  | Low | 164 (22.2%) | 1035 (29.1%) | 1199 (27.9%) |  |
|  | Med | 429 (58.0%) | 1940 (54.5%) | 2369 (55.1%) |  |
|  | Missing/Unknown | 24 (3.2%) | 73 (2.1%) | 97 (2.3%) |  |
| **Tumor size in the largest dimension-cm, Median (IQR)** |  | 1.8 (1.4-2.5) | 1.5 (1.2-2.0) | 1.5 (1.2-2.0) | <0.001 |
| **Tumor size group** | ≤2 cm | 461 (62.3%) | 2785 (78.2%) | 3246 (75.5%) | <0.001 |
|  | >2 cm | 279 (37.7%) | 775 (21.8%) | 1054 (24.5%) |  |
| **ER** | Negative | 2 (0.3%) | 12 (0.3%) | 14 (0.3%) | 1 |
|  | Positive | 738 (99.7%) | 3548 (99.7%) | 4286 (99.7%) |  |
| **PR** | Negative | 72 (9.7%) | 278 (7.8%) | 350 (8.1%) | 0.208 |
|  | Positive | 663 (89.6%) | 3253 (91.4%) | 3916 (91.1%) |  |
|  | Missing/Unknown | 5 (0.7%) | 29 (0.8%) | 34 (0.8%) |  |

Note: Risk labels were generated by the M3T model. *P* values were calculated using the chi-square test for categorical variables and the Mann–Whitney U test for continuous variables.
