# Supplementary material for: Development and Validation of a Multimodal–Multitask Deep Learning Approach for Estimating Late Distant Recurrence Risk in HR-Positive Early Breast Cancer
Source: Cancer Res Commun. 2026 Jul 31;6(7):1825–35. doi: 10.1158/2767-9764.CRC-26-0362 (PMC13425195; doi:10.1158/2767-9764.CRC-26-0362)
Supplement: Supplementary Table 12 — Predictive performance comparison of Clarity models with Breast Cancer Index (BCI). [file crc-26-0362_supplementary_table_12_suppst12.docx]

**Supplementary Table 12. Predictive performance comparison of Clarity models with Breast Cancer Index (BCI).**

| **Model** | **Risk Group** | **No. of Patients** | **Absolute Benefit (%)** | **HR (95% CI)** | ***P* value** |
| --- | --- | --- | --- | --- | --- |
| **BCI** | **Low** | 1207 | 1.4 | 0.59  (0.33–1.03) | 0.06 |
|  | **High** | 971 | 3.1 | 0.68  (0.42–1.12) | 0.13 |
| **MI Clarity** | | | | | |
| **Image-only** | **Low** | 1118 | 0.90 | 0.581  (0.271- 1.247) | 0.159 |
|  | **High** | 1153 | 3.3 | 0.670  (0.442–1.017) | 0.058 |
| **Multimodal** | **Low** | 1135 | 0.87 | 0.575  (0.247–1.342) | 0.196 |
|  | **High** | 1136 | 3.69 | 0.644  (0.43–0.965) | 0.032 |
| **Multimodal-multitask** | **Low** | 1138 | 0.49 | 0.664  (0.266–1.659) | 0.378 |
|  | **High** | 1133 | 4.09 | 0.614  (0.413–0.913) | 0.015 |
