# Supplementary material for: Development and Validation of a Multimodal–Multitask Deep Learning Approach for Estimating Late Distant Recurrence Risk in HR-Positive Early Breast Cancer
Source: Cancer Res Commun. 2026 Jul 31;6(7):1825–35. doi: 10.1158/2767-9764.CRC-26-0362 (PMC13425195; doi:10.1158/2767-9764.CRC-26-0362)
Supplement: Supplementary Table 13 — Distribution of model predicted risk groups by pathological node status across different models. [file crc-26-0362_supplementary_table_13_suppst13.docx]

**Supplementary Table 13. Distribution of model predicted risk groups by pathological node status across different models.**

| **Model** | **Pathological node status** | **Low-risk (No.)** | **High-risk (No.)** | **Total** | **Low-risk (%)** | **High-risk (%)** |
| --- | --- | --- | --- | --- | --- | --- |
| **Image-only** | Negative | 769 | 596 | 1365 | 56.40% | 43.60% |
|  | Positive | 349 | 557 | 906 | 38.50% | 61.50% |
| **Multimodal** | Negative | 994 | 371 | 1365 | 72.90% | 27.20% |
|  | Positive | 141 | 765 | 906 | 15.60% | 84.40% |
| **Multimodal-multitask** | Negative | 969 | 396 | 1365 | 71.00% | 29.00% |
|  | Positive | 169 | 737 | 906 | 18.70% | 81.30% |
